# Supplementary material for: Stem Leydig cells support macrophage immunological homeostasis through mitochondrial transfer in mice
Source: Nat Commun. 2024 Mar 8;15:2120. doi: 10.1038/s41467-024-46190-2 (PMC10924100; doi:10.1038/s41467-024-46190-2)
Supplement: Supplementary file 1 — Supplementary Information [file 41467_2024_46190_MOESM1_ESM.pdf]

Supplementary Information

Stem Leydig cells support macrophage immunological homeostasis through mitochondrial transfer in mice

Ani Chi, Bicheng Yang, Hao Dai, Xinyu Li, Jiahui Mo, Yong Gao, Zhihong, Chen, Xin Feng, Menghui Ma, Yanqing Li, Chao Yang, Jie Liu, Hanchao Liu, Zhengqing Wang, Xuetao Shi, Chunhua Deng, Min Zhang

Xuetao Shi (shxt@scut.edu.cn), Chunhua Deng (dengchh@mail.sysu.edu.cn), Min Zhang (zhangm287@mail.sysu.edu.cn)

Contents for Supporting Information

Supplementary Figures

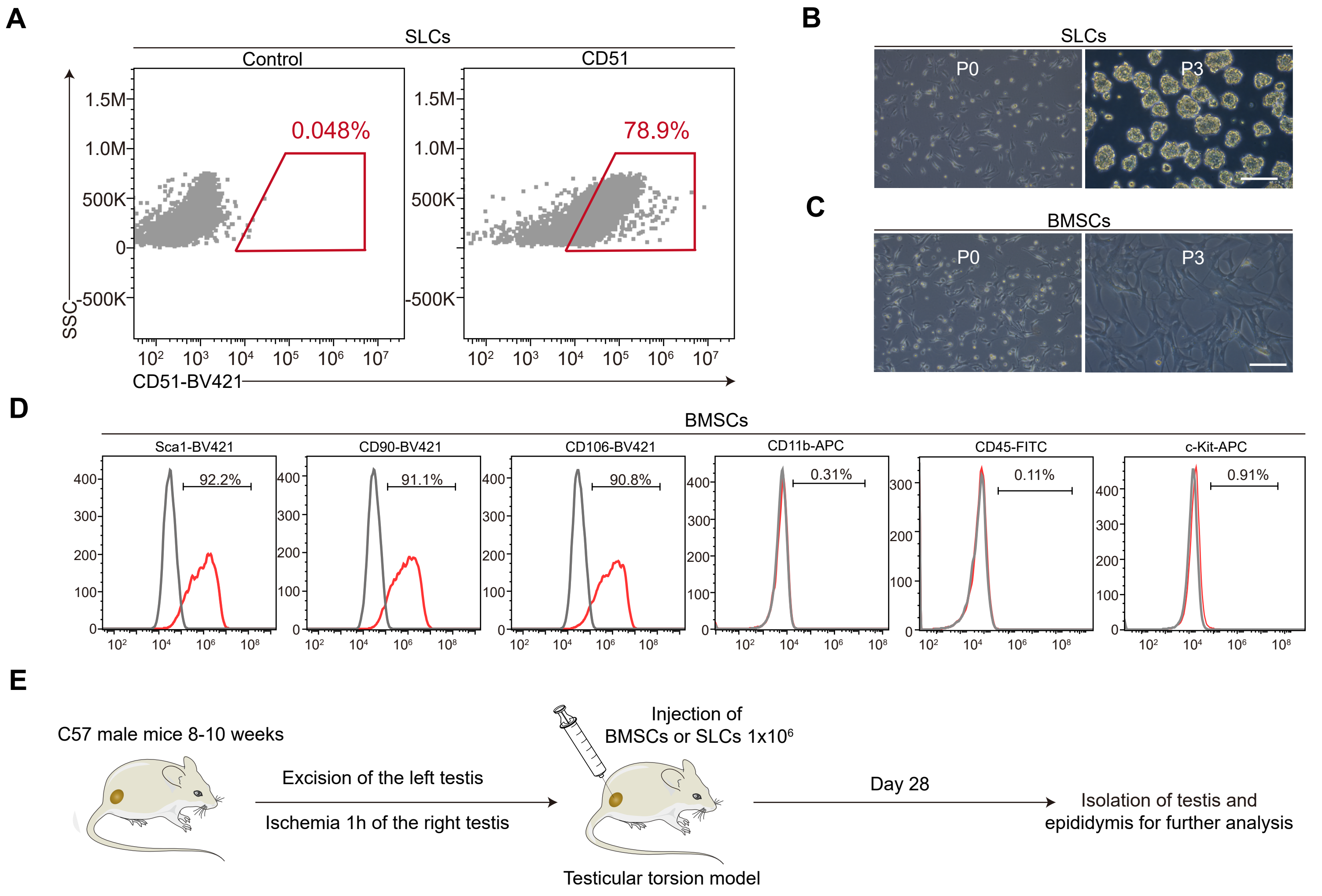

F

		Sham				Saline				BMSCs				SLCs			
Mouse numbers		1	2	3	4	1	2	3	4	1	2	3	4	1	2	3	4
Sprem counts (million/mL)		2830	2950	2870	2130	500	510	610	670	760	740	780	670	1020	1180	1160	1200
Sperm motility	PR(%)	14.9%	13.8%	13.5%	14.2%	5.3%	3.9%	3.0%	1.7%	9.1%	7.4%	11.1%	4.3%	11.0%	8.0%	10.2%	5.9%
	NR(%)	27.6%	29.2%	31.5%	27.4%	7.1%	5.3%	12.7%	1.7%	15.4%	17.4%	13.1%	16.1%	24.0%	25.3%	29.9%	20.9%
	IM(%)	57.5%	56.9%	55.1%	58.5%	87.6%	90.8%	84.3%	96.6%	75.5%	75.2%	75.8%	79.6%	65.0%	66.7%	59.9%	73.2%

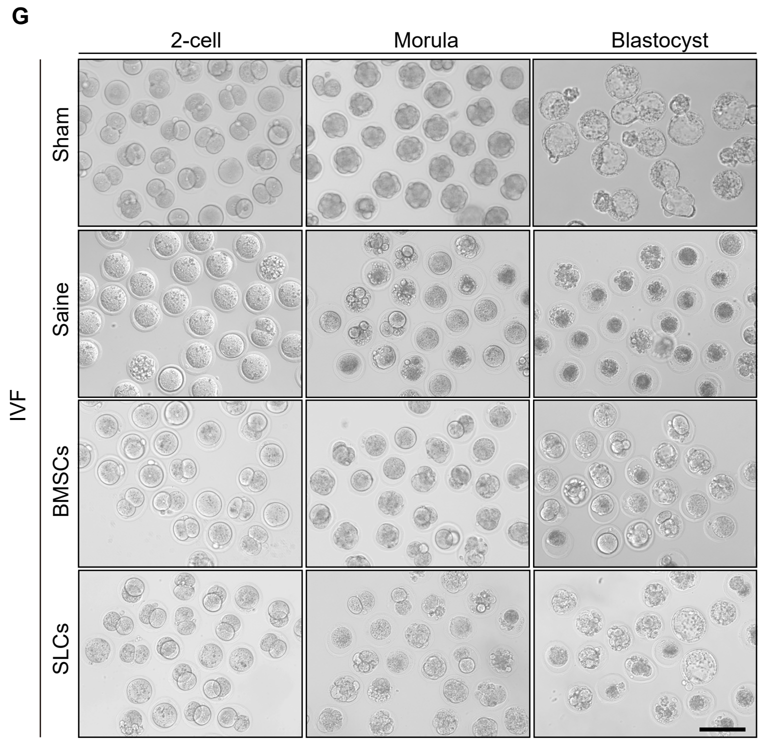

Supplementary Fig. 1| Isolation and phenotypic analysis of BMSCs and SLCs. a, Representative flow cytometry profiles showing the isolation of $CD51^+$ cells from the testes of mice at postnatal day 7. **b**, Micrographs of primary (P0, 2 days after plating) and P3 (15 days after plating) cells in each population derived from the SLCs. Scale bar, 100 μm. **c**, Micrographs of primary (P0, 2 days after plating) and P3 (15 days after plating) cells in each population derived from the BMSCs. Scale bar, 100 μm. **d**, Representative flow cytometry profiles showing the expression of some MSC-associated surface markers on BMSCs. **e**, Schematic illustration of the experimental and analysis workflow. **f**, Sperm counts and sperm motility in the sham saline, BMSCs and SLCs groups. PR: progressive motile, NR: nonprogressive motile, IM: immotilitile. **g**, Bright field diagram of the 2-cell, morula, and blastocyst stages among the sham, saline, BMSCs, SLCs groups. Scale bar, 200 μm.

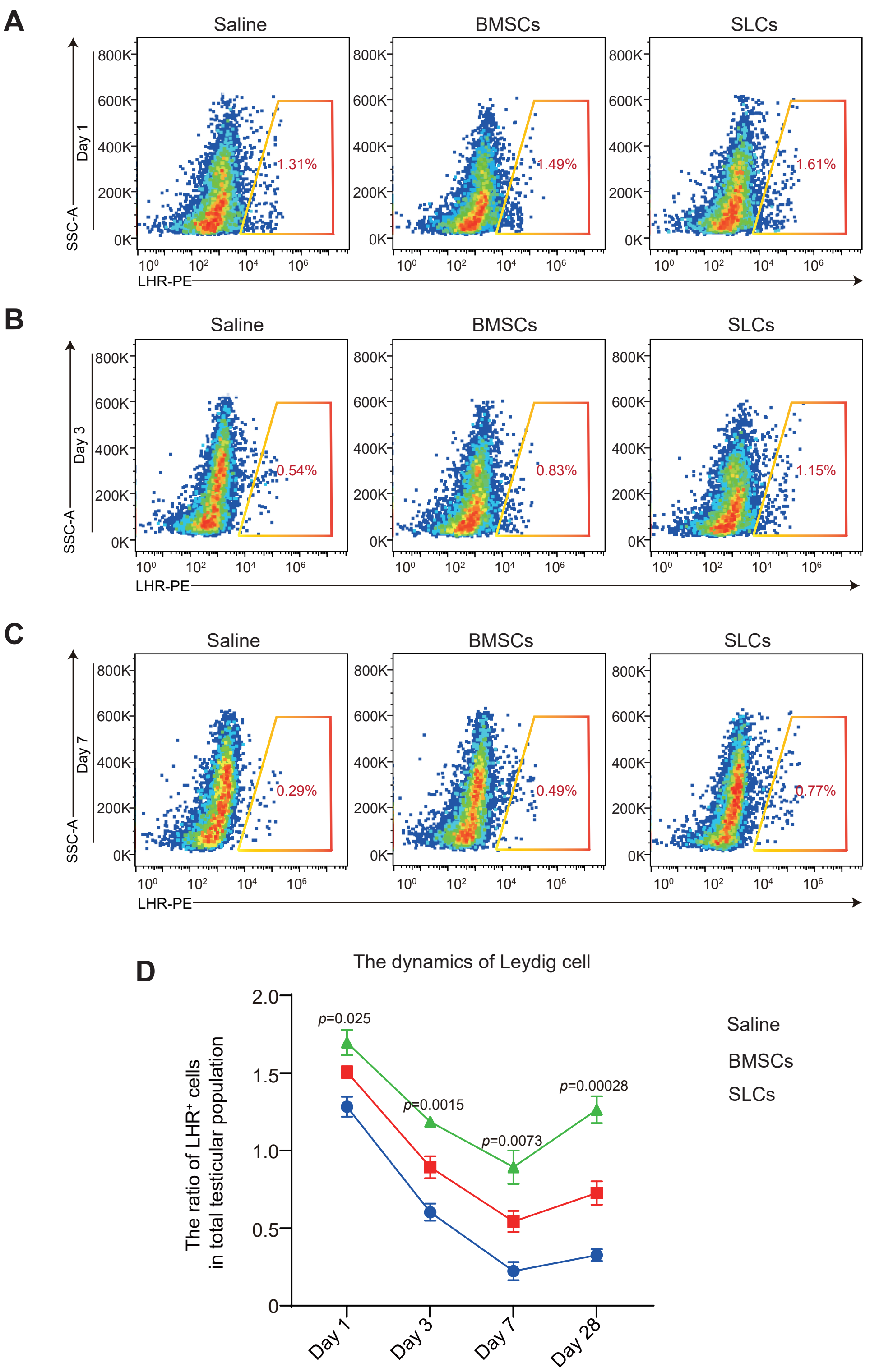
A
Saline
BMSCs
SLCs
Day 1
SSC-A
LHR-PE
1.31%
1.49%
1.61%
B
Day 3
0.54%
0.83%
1.15%
C
Day 7
0.29%
0.49%
0.77%
D
The dynamics of Leydig cell
The ratio of LHR+ cells in total testicular population
p=0.025
p=0.0015
p=0.0073
p=0.00028
Day 1
Day 3
Day 7
Day 28
Saline
BMSCs
SLCs

Supplementary Fig. 2| Transplanted SLCs promote the regeneration of LCs. a-c, Flow cytometry for detecting the percentage of LHR^+ cells among the total testicular cells in the saline, BMSCs and SLCs groups on days 1, 3 and 7. **d**, The dynamic ratio of LCs among the total testicular cells in the saline, BMSCs and SLCs groups on days 1, 3, 7 and 28. n = 3 biological replicates for each group. One-way ANOVA was used. Source data are provided as a Source Data file.

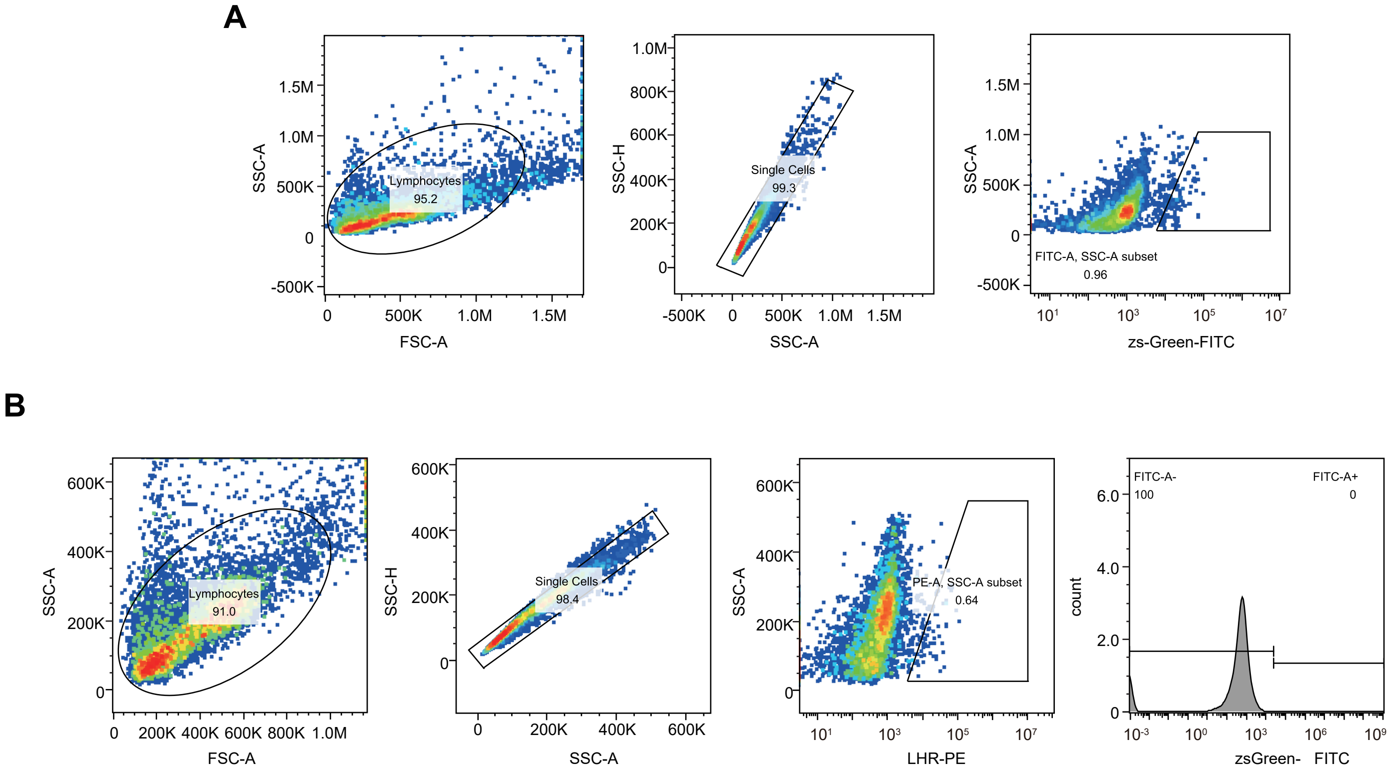
A
Lymphocytes
95.2
SSC-A
FSC-A
Single Cells
99.3
SSC-H
SSC-A
FITC-A, SSC-A subset
0.96
SSC-A
zs-Green-FITC
B
Lymphocytes
91.0
SSC-A
FSC-A
Single Cells
98.4
SSC-H
SSC-A
PE-A, SSC-A subset
0.64
SSC-A
LHR-PE
FITC-A-
100
FITC-A+
0
count
zsGreen- FITC

Supplementary Fig. 3| Gate selection for BMSCs^{ZsGreen}, SLCs^{ZsGreen} and LCs a, Representative FACS gating scheme for BMSCs^{ZsGreen} or SLCs^{ZsGreen} in total testicular cells. **b**, Representative FACS gating scheme for the LHR^{+} zsGreen^{+} or LHR^{+} zsGreen^{-} cells among total testicular cells.

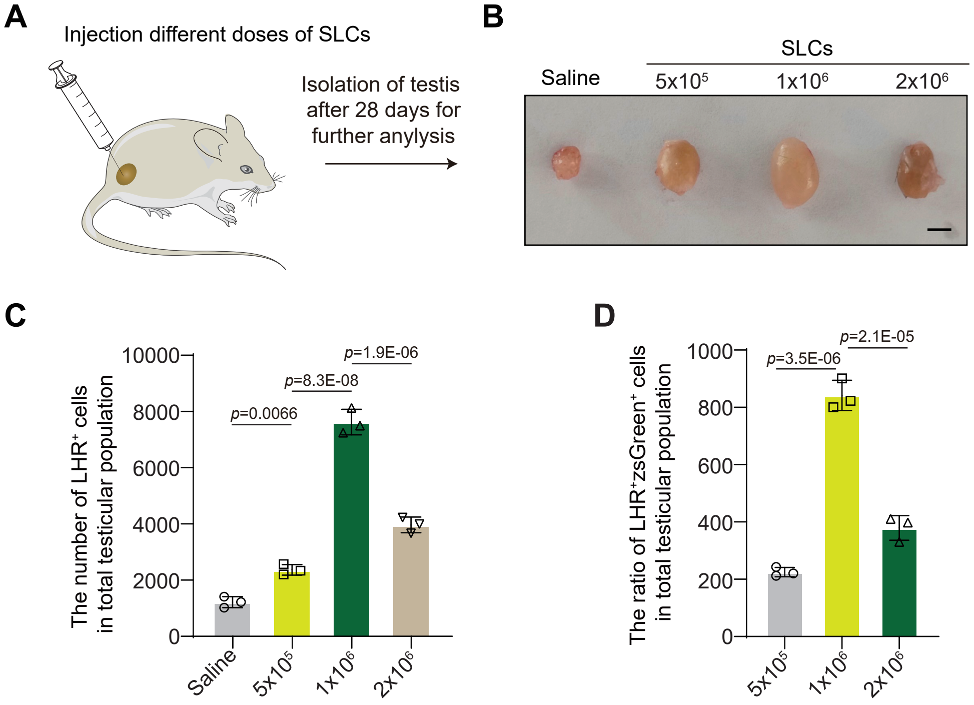
A
Injection different doses of SLCs
Isolation of testis after 28 days for further anylysis
B
SLCs
Saline
5x10^5
1x10^6
2x10^6
C
The number of LHR+ cells in total testicular population
10000
8000
6000
4000
2000
0
p=0.0066
p=8.3E-08
p=1.9E-06
Saline
5x10^5
1x10^6
2x10^6
D
The ratio of LHR+zsGreen+ cells in total testicular population
1000
800
600
400
200
0
p=3.5E-06
p=2.1E-05
5x10^5
1x10^6
2x10^6

Supplementary Fig. 4| Effect of SLCs transplantation at different doses on LCs recovery. a, Experimental timeline. This timeline represents the different doses of SLCs transplantation. **b**, Bright field diagram of testicular size (scale bar, 2 mm) of testis samples obtained from the saline, 5×10^5, 1×10^6, 2×10^6 SLCs treated groups at day 28 after testicular torsion. **c-d**, Quantification of the number of LHR^+ or $LHR^+zsGreen^+$ cells among the total testicular cells in different groups. The data are presented as the means ± SDs. n = 3 biological replicates for each group. One-way ANOVA was used. Source data are provided as a Source Data file.

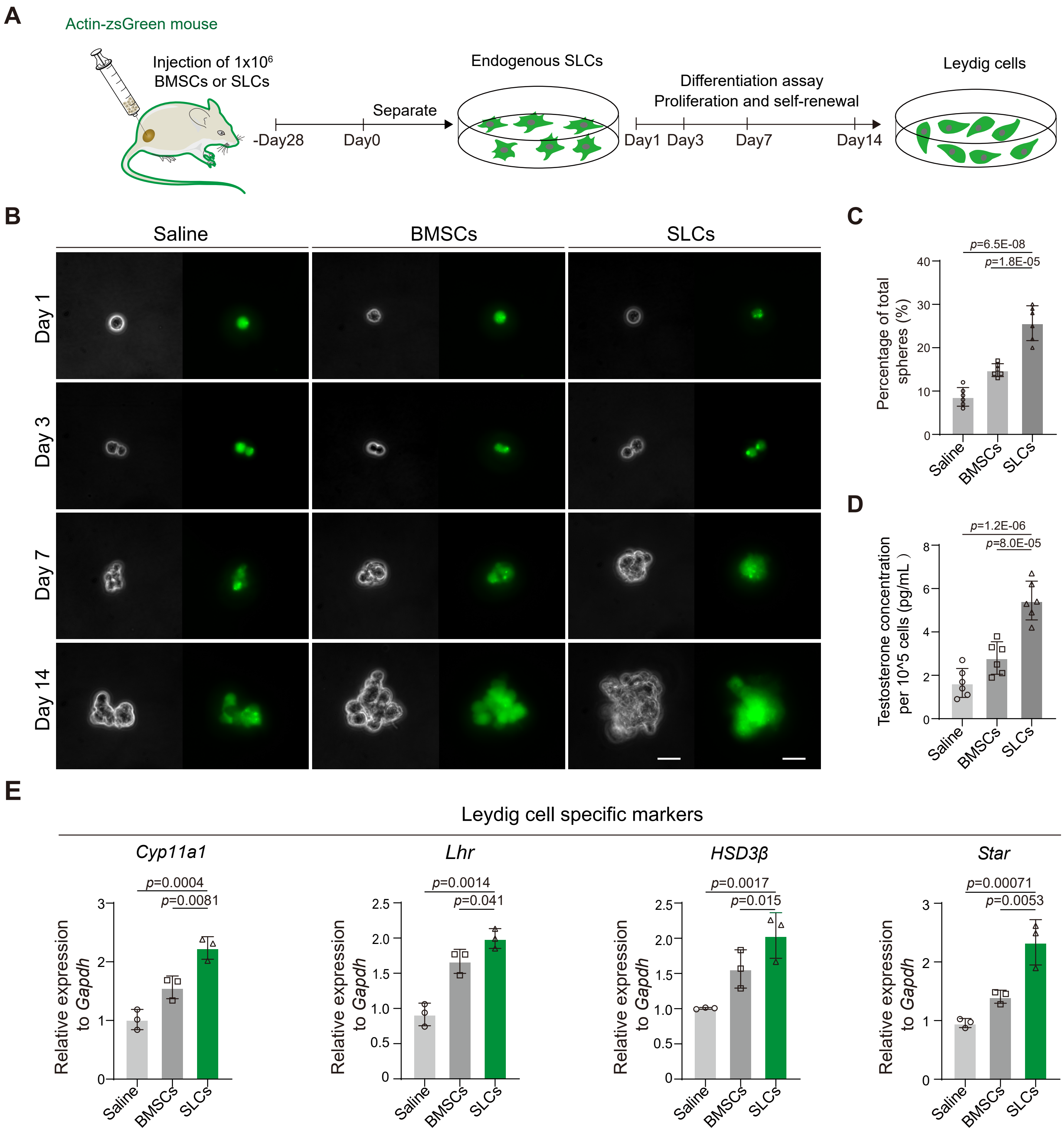
A
Actin-zsGreen mouse
Injection of 1x10^6 BMSCs or SLCs
-Day28
Day0
Separate
Endogenous SLCs
Differentiation assay
Proliferation and self-renewal
Day1 Day3 Day7 Day14
Leydig cells
B
Saline
BMSCs
SLCs
Day 1
Day 3
Day 7
Day 14
C
Percentage of total spheres (%)
p=6.5E-08
p=1.8E-05
Saline BMSCs SLCs
D
Testosterone concentration per 10^5 cells (pg/mL)
p=1.2E-06
p=8.0E-05
Saline BMSCs SLCs
E
Leydig cell specific markers
Cyp11a1
p=0.0004
p=0.0081
Lhr
p=0.0014
p=0.041
HSD3β
p=0.0017
p=0.015
Star
p=0.00071
p=0.0053
Relative expression to Gapdh
Saline BMSCs SLCs

Supplementary Fig. 5| Effect of SLCs transplantation on the differentiation of endogenous SLCs into LCs in vitro. **a**, Schematic of the experimental procedure for isolating primary endogenous SLCs and inducing differentiation into LCs. **b**, Representative bright field images show clonal spheres from single cells. Scale bar, 100 μm. **c**, Comparison of sphere formation in wells containing a single cell showing the frequency of sphere formation. The data are expressed as the means ± SDs. **d**, Quantification of testosterone levels in the supernatant of medium during induced differentiation of primary endogenous SLCs from different groups. The data are presented as the means ± SDs. n = 6 biological replicates for each group. One-way ANOVA was used. **e**, RT–PCR analysis of the relative mRNA expression of testosterone production-related genes on day 14 during the induced differentiation of primary endogenous SLCs from different groups. Source data are provided as a Source Data file.

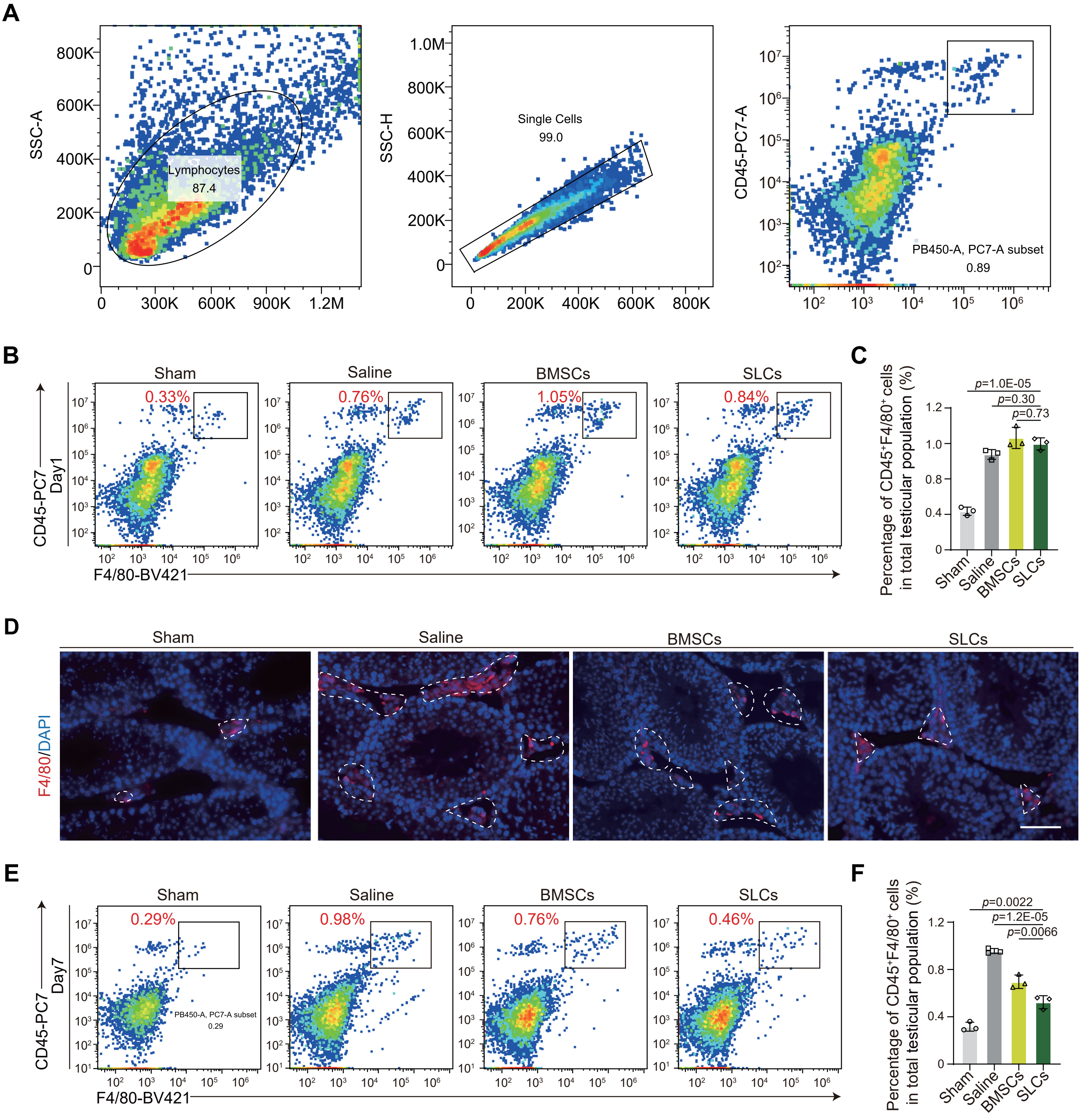
A
SSC-A
Lymphocytes
87.4
SSC-H
Single Cells
99.0
CD45-PC7-A
PB450-A, PC7-A subset
0.89
B
Sham
Saline
BMSCs
SLCs
0.33%
0.76%
1.05%
0.84%
CD45-PC7
Day1
F4/80-BV421
C
Percentage of CD45+F4/80+ cells in total testicular population (%)
p=1.0E-05
p=0.30
p=0.73
D
F4/80/DAPI
E
0.29%
0.98%
0.76%
0.46%
PB450-A, PC7-A subset
0.29
Day7
F
p=0.0022
p=1.2E-05
p=0.0066

Supplementary Fig. 6| Transplantation of SLCs significantly reduces the total number of macrophages in the testis after testicular torsion. **a**, Representative FACS gating scheme for the flow cytometry experiment to study the ratio of $CD45^+F4/80^+$ macrophages in vivo. **b**, Flow cytometry for detecting the percentage of $CD45^+F4/80^+$ macrophages among total testicular cells on day 1. **c**, Quantitative analysis of the percentage of $CD45^+F4/80^+$ macrophages in the saline, BMSCs and SLCs groups on day 1. The data are presented as the means ± SDs, n=3 biological replicates for each group. One-way ANOVA was used. **d**, Immunostaining of F4/80 at 3 days after testicular torsion in paraffin sections. Scale bar, 50 μm. **e**, Flow cytometry for detecting the percentage of $CD45^+F4/80^+$ macrophages among the total testicular cells on day 7. **f**, Quantitative analysis of the percentage of $CD45^+F4/80^+$ macrophages in the saline, BMSCs and SLCs groups on day 7. The data are presented as the means ± SDs. n=3 biological replicates for each group. One-way ANOVA was used. Source data are provided as a Source Data file.

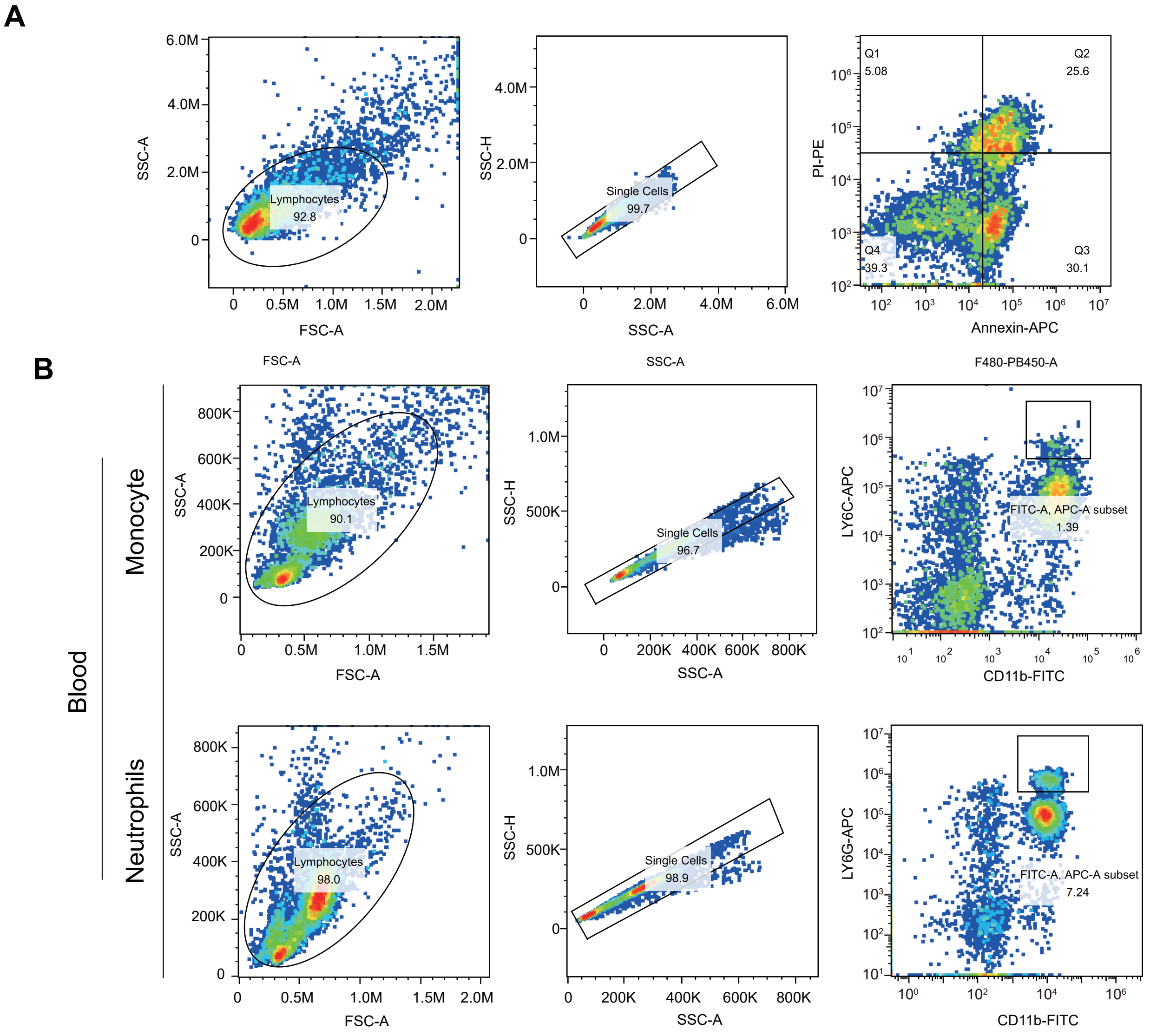
A
Lymphocytes
92.8
SSC-A
FSC-A
Single Cells
99.7
SSC-H
SSC-A
Q1
5.08
Q2
25.6
Q4
39.3
Q3
30.1
PI-PE
Annexin-APC
B
FSC-A
SSC-A
F480-PB450-A
Blood
Monocyte
Neutrophils
Lymphocytes
90.1
Single Cells
96.7
FITC-A, APC-A subset
1.39
LY6C-APC
CD11b-FITC
Lymphocytes
98.0
Single Cells
98.9
FITC-A, APC-A subset
7.24
LY6G-APC
CD11b-FITC

Supplementary Fig. 7| Gating strategy for flow cytometry analysis. a, Representative FACS gating scheme for the Annexin V-APC apoptosis assay. **b**, Representative FACS gating scheme for monocyte and neutrophil gate selection in blood cells.

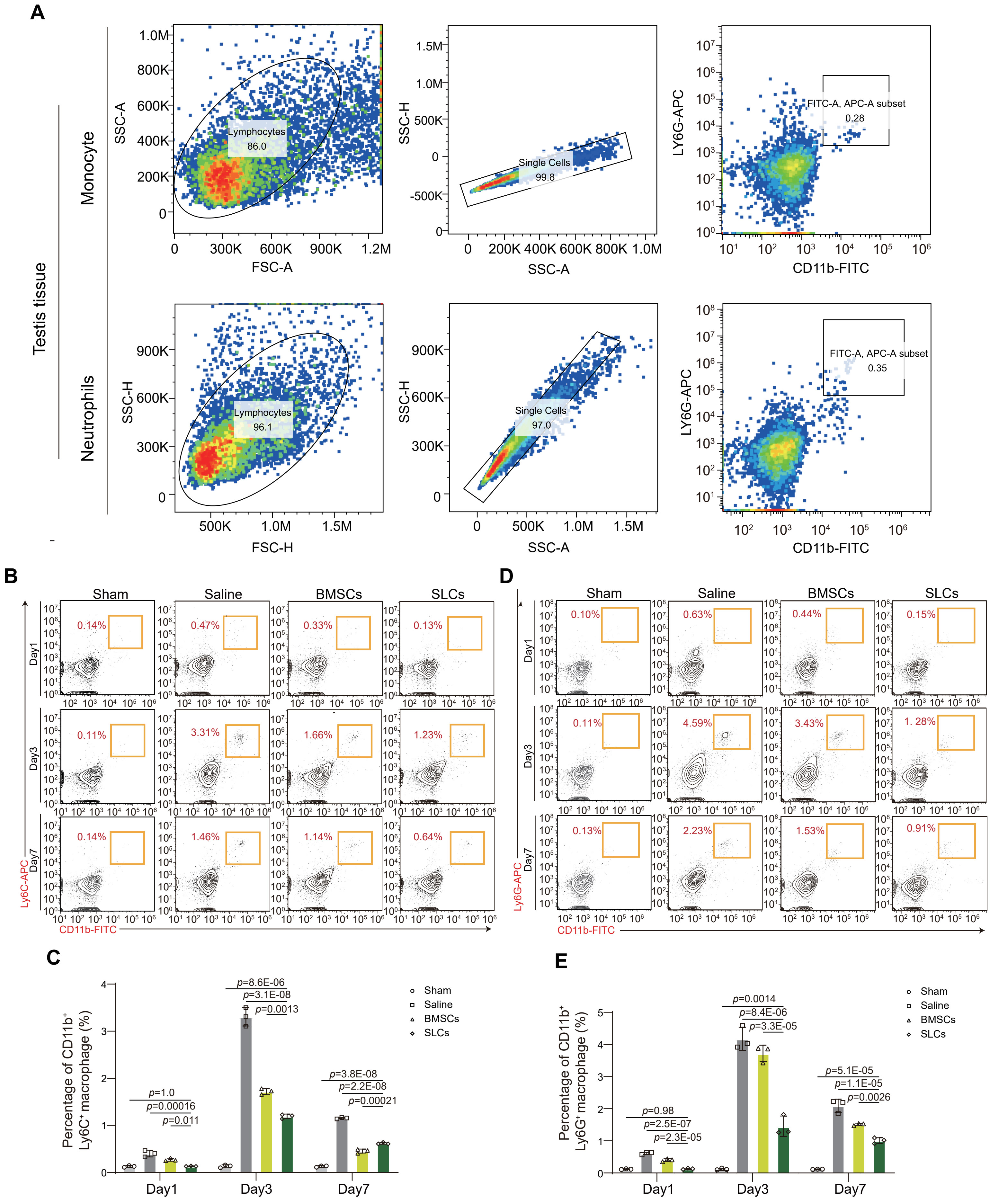
A
Testis tissue
Monocyte
Neutrophils
SSC-A
FSC-A
Lymphocytes
86.0
SSC-H
Single Cells
99.8
LY6G-APC
CD11b-FITC
FITC-A, APC-A subset
0.28
FSC-H
Lymphocytes
96.1
Single Cells
97.0
FITC-A, APC-A subset
0.35
B
Sham
Saline
BMSCs
SLCs
Day1
Day3
Day7
0.14%
0.47%
0.33%
0.13%
0.11%
3.31%
1.66%
1.23%
0.14%
1.46%
1.14%
0.64%
Ly6C-APC
CD11b-FITC
D
0.10%
0.63%
0.44%
0.15%
0.11%
4.59%
3.43%
1. 28%
0.13%
2.23%
1.53%
0.91%
Ly6G-APC
C
Percentage of CD11b⁺ Ly6C⁺ macrophage (%)
p=1.0
p=0.00016
p=0.011
p=8.6E-06
p=3.1E-08
p=0.0013
p=3.8E-08
p=2.2E-08
p=0.00021
Sham
Saline
BMSCs
SLCs
E
Percentage of CD11b⁺ Ly6G⁺ macrophage (%)
p=0.98
p=2.5E-07
p=2.3E-05
p=0.0014
p=8.4E-06
p=3.3E-05
p=5.1E-05
p=1.1E-05
p=0.0026

Supplementary Fig. 8| SLCs transplantation suppresses monocyte and neutrophil infiltration into the testis after testicular torsion. a, Representative FACS gating scheme for monocyte and neutrophil gate selection in the testis. **b**, Representative flow cytometry profiles showing the percentage of $CD11b^{+}Ly6C^{+}$ cells in the testes in each group at 1, 3, and 7 days after cell transplantation. **c**, Quantification of the percentage of $CD11b^{+}Ly6C^{+}$ cells in the testes in each group at 1, 3, and 7 days. The data are presented as the means ± SDs. n=3 biological replicates for each group. One-way ANOVA was used. **d**, Representative flow cytometry profiles showing the percentage of $CD11b^{+}Ly6G^{+}$ cells in the testes of each group at 1, 3, and 7 days after cell transplantation. **e**, Quantitative analysis of the percentage of $CD11b^{+}Ly6G^{+}$ cells in the testes of each group at 1, 3, and 7 days. The data are presented as the means ± SDs, n=3 biological replicates for each group. One-way ANOVA was used. Source data are provided as a Source Data file.

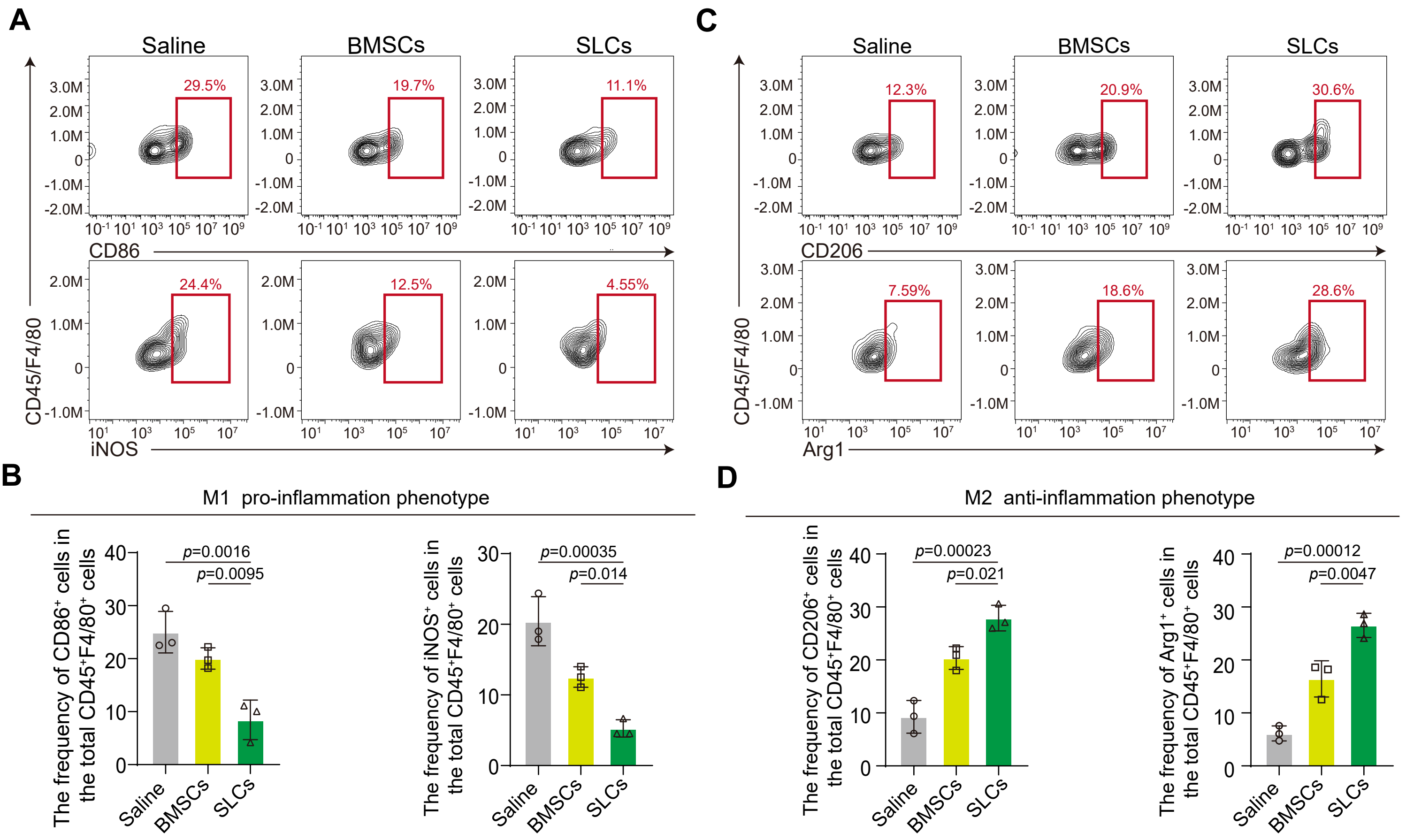
A
Saline
BMSCs
SLCs
29.5%
19.7%
11.1%
24.4%
12.5%
4.55%
CD86
iNOS
CD45/F4/80
C
12.3%
20.9%
30.6%
7.59%
18.6%
28.6%
CD206
Arg1
B
M1 pro-inflammation phenotype
The frequency of CD86⁺ cells in the total CD45⁺F4/80⁺ cells
p=0.0016
p=0.0095
The frequency of iNOS⁺ cells in the total CD45⁺F4/80⁺ cells
p=0.00035
p=0.014
D
M2 anti-inflammation phenotype
The frequency of CD206⁺ cells in the total CD45⁺F4/80⁺ cells
p=0.00023
p=0.021
The frequency of Arg1⁺ cells in the total CD45⁺F4/80⁺ cells
p=0.00012
p=0.0047

Supplementary Fig. 9| SLCs reshaped the inflammatory properties of macrophages. a, Representative flow cytometry profiles showing CD86 and iNOS among total $CD45^+F4/80^+$ macrophages in testes on day 7. **b**, Quantitative analysis of the percentage of M1 phenotype-specific markers (CD86 and iNOS) among total $CD45^+F4/80^+$ macrophages in testes on day 7. The data are presented as the means ± SDs. n=3 biological replicates for each group. One-way ANOVA was used. **c**, Representative flow cytometry profiles showing CD206 and Arg1 on $CD45^+F4/80^+$ macrophages in testes on day 7. **d**, Quantitative analysis of the percentage of the M2 phenotype-specific markers (CD206 and Arg1) on $CD45^+F4/80^+$ macrophages in testes on day 7. The data are presented as the means ± SDs. n=3 biological replicates for each group. One-way ANOVA was used. Source data are provided as a Source Data file.

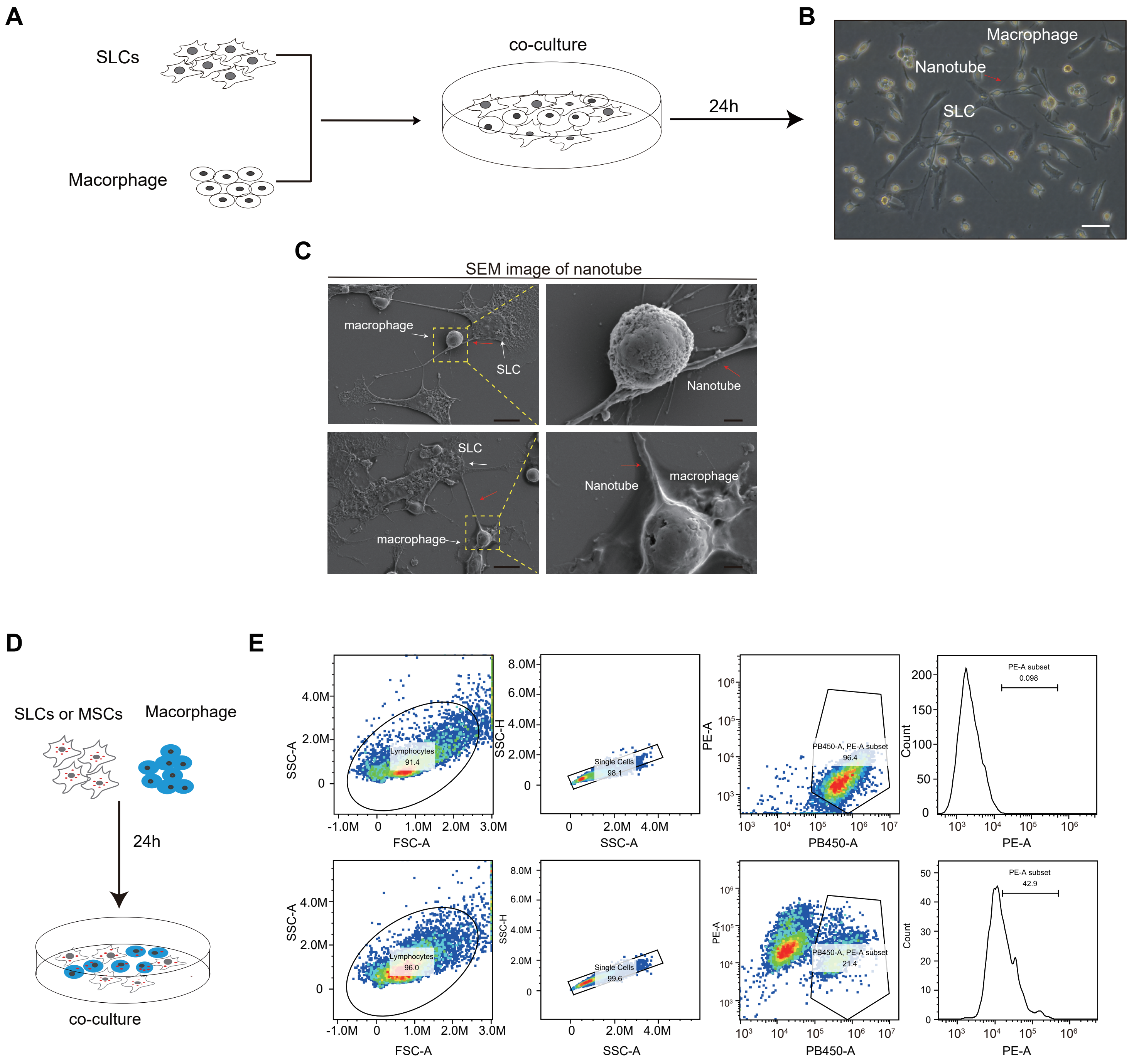
A
SLCs
Macorphage
co-culture
24h
B
Macrophage
Nanotube
SLC
C
SEM image of nanotube
macrophage
SLC
Nanotube
SLC
macrophage
Nanotube
macrophage
D
SLCs or MSCs
Macorphage
24h
co-culture
E
Lymphocytes
91.4
SSC-A
FSC-A
Single Cells
98.1
SSC-H
SSC-A
PB450-A, PE-A subset
96.4
PE-A
PB450-A
PE-A subset
0.098
Count
PE-A
Lymphocytes
96.0
Single Cells
99.6
PB450-A, PE-A subset
21.4
PE-A subset
42.9

Supplementary Fig. 10| Gating strategy for flow cytometry analysis. **a**, Schematic of the experimental procedure for the coculture of SLCs with macrophages **b**, Micrographs of macrophage coculture with SLCs. **c**, SEM images showing nanotubes (red arrow) between SLCs and macrophages. Scale bar, 1 μm, the larger scale bar 10 μm. **d**, Schematic of the experimental procedure for the coculture of SLCs with macrophages, macrophages stained with CellTrace Violet, and SLCs labeled with MitoTracker Red CMXRos. **e**, Representative FACS gating scheme for the flow cytometry experiment to study the ratio of mitochondria in $CD45^{+}F4/80^{+}$ macrophages in vivo. A higher intensity of MITO-DsRed corresponds to macrophages that have accepted excess mitochondria from transplanted BMSCs or SLCs.

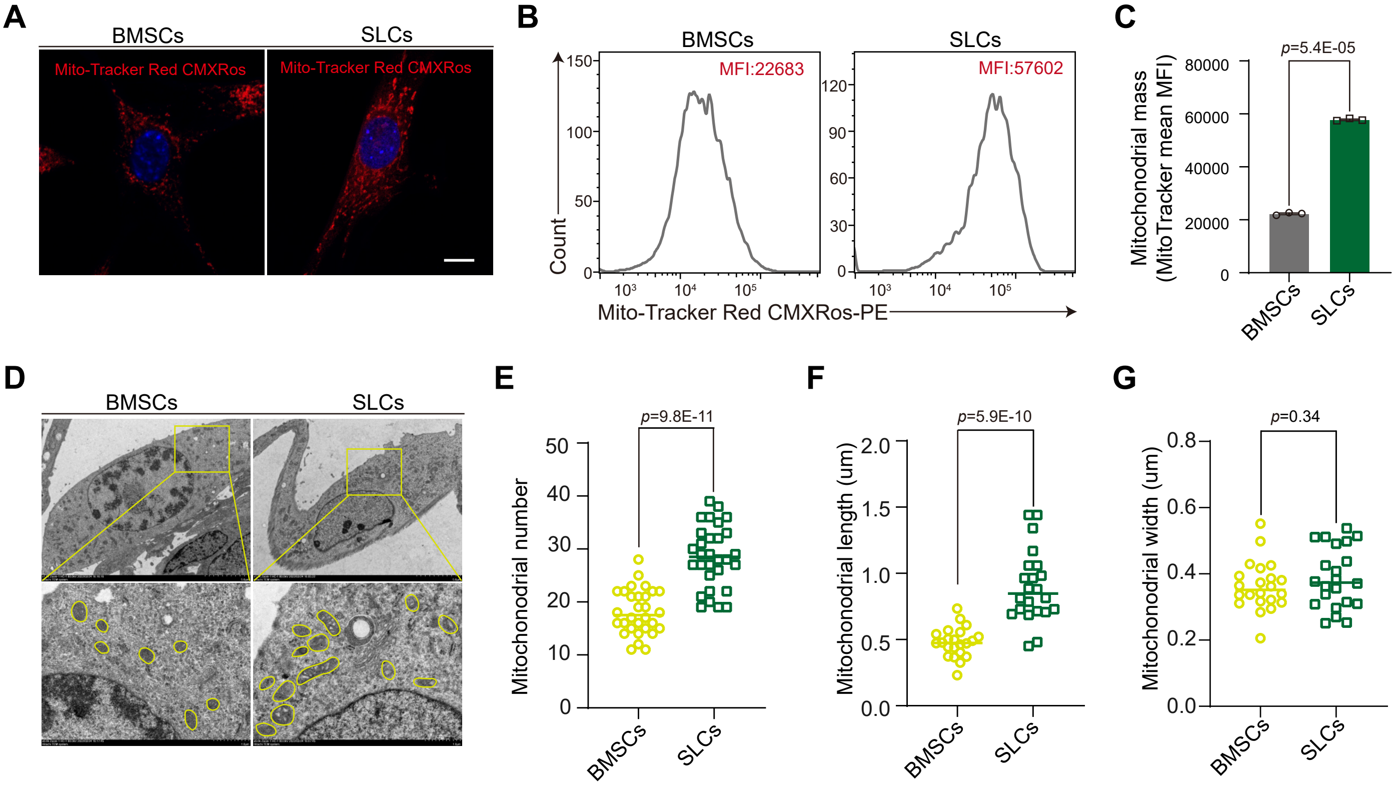

A
BMSCs
SLCs
Mito-Tracker Red CMXRos
Mito-Tracker Red CMXRos
B
BMSCs
SLCs
MFI:22683
MFI:57602
Count
Mito-Tracker Red CMXRos-PE
C
p=5.4E-05
Mitochondrial mass (MitoTracker mean MFI)
BMSCs
SLCs
D
BMSCs
SLCs
E
p=9.8E-11
Mitochonodrial number
BMSCs
SLCs
F
p=5.9E-10
Mitochonodrial length (um)
BMSCs
SLCs
G
p=0.34
Mitochonodrial width (um)
BMSCs
SLCs

Supplementary Fig. 11| The mitochondrial mass of SLCs and BMSCs. **a**, Representative confocal microscopy of BMSCs and SLCs mitochondria (red) labeled with MitoTracker Red CMXRos. **b**, Representative flow cytometry profiles showing the mitochondrial mass in BMSCs and SLCs. **c**, Quantitative analysis of mitochondrial mass by median fluorescence intensity (MFI) for BMSCs and SLCs. The data are presented as the means ± SDs, n=3 biological replicates for each group. Unpaired-tailed Student's t test was used. **d**, Representative electron microscopy images of BMSCs and SLCs. The red circle indicates the mitochondria. **e-g**, Quantitative analysis of mitochondrial number, mitochondrial width, and mitochondrial length. The data are presented as the means ± SDs. n = 30 cells examined over 3 independent experiments. Unpaired two tailed Student's t test was used. Source data are provided as a Source Data file.

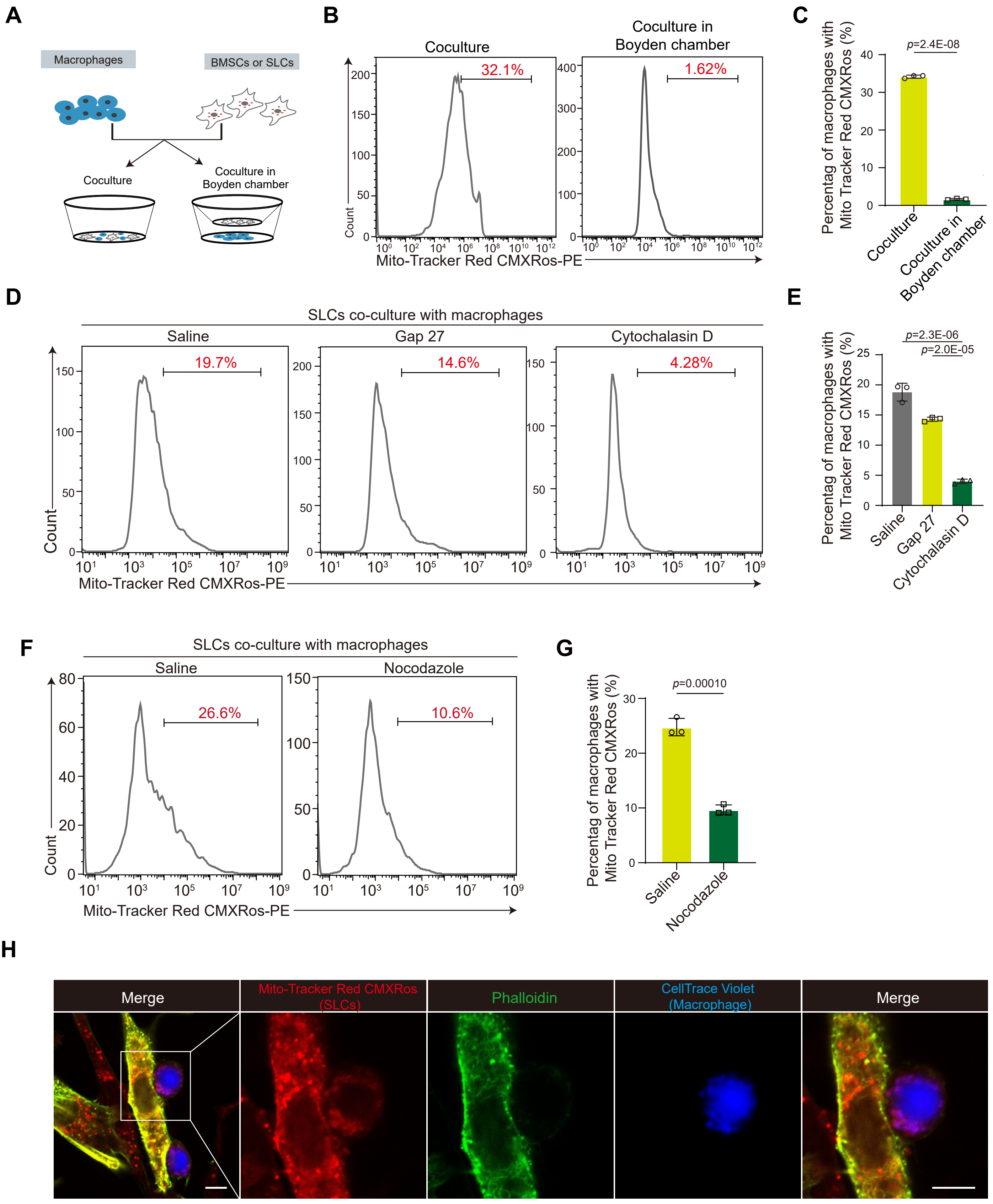
A
Macrophages
BMSCs or SLCs
Coculture
Coculture in Boyden chamber
B
Coculture
Coculture in Boyden chamber
32.1%
1.62%
Count
Mito-Tracker Red CMXRos-PE
C
p=2.4E-08
Percentag of macrophages with Mito Tracker Red CMXRos (%)
Coculture
Coculture in Boyden chamber
D
SLCs co-culture with macrophages
Saline
Gap 27
Cytochalasin D
19.7%
14.6%
4.28%
Count
Mito-Tracker Red CMXRos-PE
E
p=2.3E-06
p=2.0E-05
Percentag of macrophages with Mito Tracker Red CMXRos (%)
Saline
Gap 27
Cytochalasin D
F
SLCs co-culture with macrophages
Saline
Nocodazole
26.6%
10.6%
Count
Mito-Tracker Red CMXRos-PE
G
p=0.00010
Percentag of macrophages with Mito Tracker Red CMXRos (%)
Saline
Nocodazole
H
Merge
Mito-Tracker Red CMXRos (SLCs)
Phalloidin
CellTrace Violet (Macrophage)
Merge

Supplementary Fig. 12| SLCs transferred mitochondria to macrophages through nanotubules. **a**, Schematic of the experiment in which SLCs were cultured with macrophages in direct contact or separated in a Boyden chamber. **b**, Representative flow cytometry profiles showing that SLCs transfer mitochondria to total macrophages in direct contact or separated in a Boyden chamber. **c**, The percentage of mitochondrial transfer to total macrophages in each group was analyzed and graphed. The data are presented as the means ± SDs, n=3 biological replicates for each group. Unpaired two tailed Student's t test was used. **d**, Representative flow cytometry profiles showing SLCs transfer of mitochondria to total macrophages with Gap27 or cytochalasin D treatment for 24 h. **e**, The percentage of mitochondrial transfer to total macrophages in each group was analyzed and graphed. The data are presented as the means ± SDs, n=3 biological replicates for each group. One-way ANOVA was used. **f**, Representative flow cytometry profiles showing SLCs transfer of mitochondria to total macrophages with nocodazole treatment for 24 h. **g**, The percentage of mitochondrial transfer to total macrophages in each group was analyzed and graphed. The data are presented as the means ± SDs, n=3 biological replicates for each group. One-way ANOVA was used. **h**, Representative confocal microscopy of macrophages stained with CellTrace Violet cocultured with SLCs labeled with MitoTracker Red CMXRos. Scale bar, 50 μm. Source data are provided as a Source Data file.

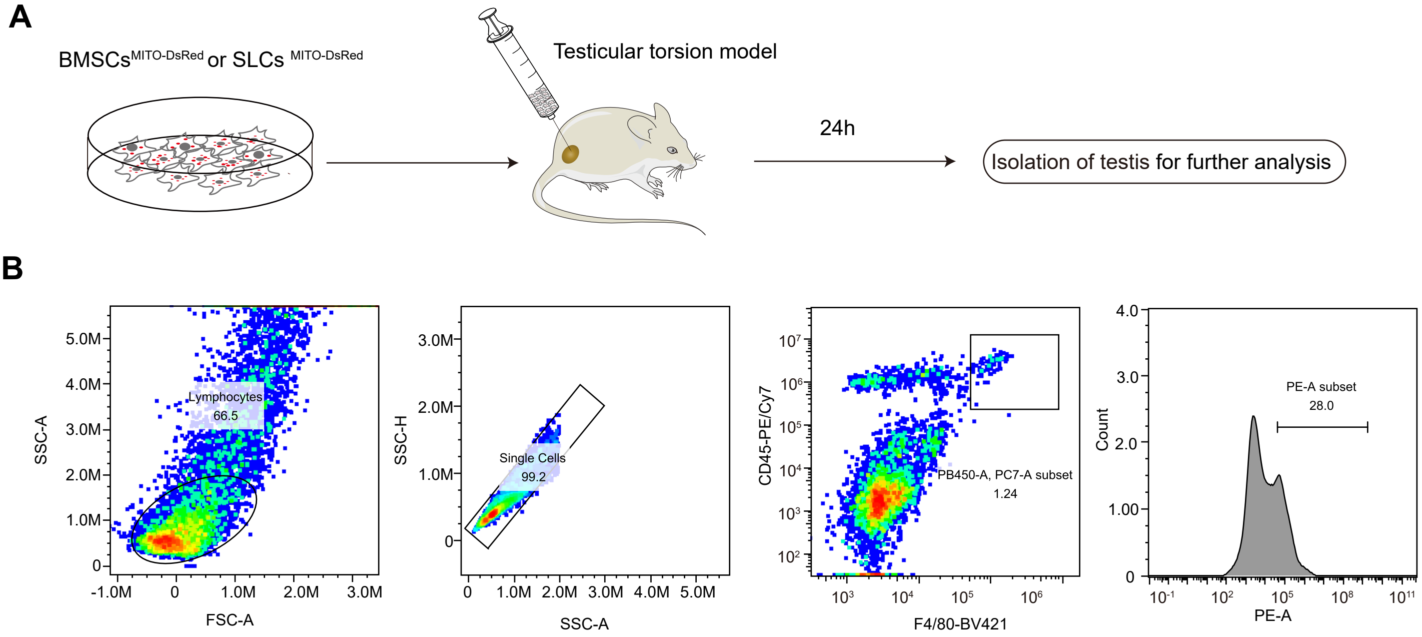
A
BMSCsMITO-DsRed or SLCs MITO-DsRed
Testicular torsion model
24h
Isolation of testis for further analysis
B
Lymphocytes
66.5
SSC-A
FSC-A
Single Cells
99.2
SSC-H
CD45-PE/Cy7
F4/80-BV421
PB450-A, PC7-A subset
1.24
PE-A subset
28.0
Count
PE-A

Supplementary Fig. 13| Gate selection for FACS. a, Schematic of the experiment used to study the ratio of mitochondria in $CD45^+F4/80^+$ macrophages in vivo. **b**, Representative FACS gating scheme for the flow cytometry experiment to study the ratio of mitochondria in $CD45^+F4/80^+$ macrophages in vivo. A higher intensity of MITO-DsRed corresponds to macrophages that have accepted excess mitochondria from transplanted BMSCs or SLCs.

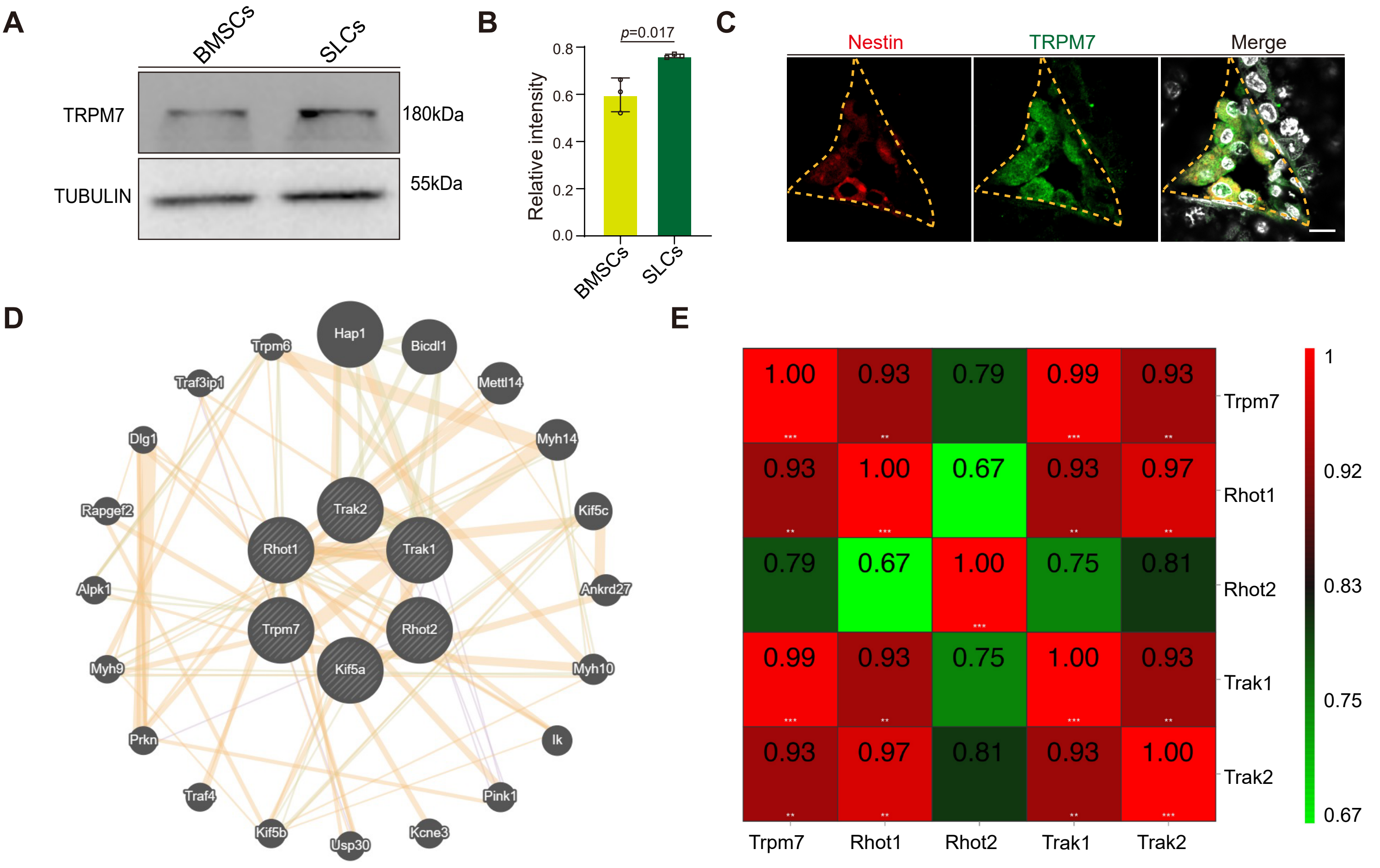
A
BMSCs
SLCs
TRPM7
180kDa
TUBULIN
55kDa
B
p=0.017
Relative intensity
0.0
0.2
0.4
0.6
0.8
BMSCs
SLCs
C
Nestin
TRPM7
Merge
D
Hap1
Bicdl1
Trpm6
Traf3ip1
Mettl14
Dlg1
Myh14
Rapgef2
Kif5c
Trak2
Rhot1
Trak1
Alpk1
Ankrd27
Trpm7
Rhot2
Kif5a
Myh9
Myh10
Prkn
Ik
Traf4
Pink1
Kif5b
Usp30
Kcne3
E
1.00 0.93 0.79 0.99 0.93 Trpm7
0.93 1.00 0.67 0.93 0.97 Rhot1
0.79 0.67 1.00 0.75 0.81 Rhot2
0.99 0.93 0.75 1.00 0.93 Trak1
0.93 0.97 0.81 0.93 1.00 Trak2
Trpm7
Rhot1
Rhot2
Trak1
Trak2
1
0.92
0.83
0.75
0.67

Supplementary Fig. 14| Correlation between TRPM7 and mitochondrial transport genes. a, Western blot analysis of Trpm7 in SLCs and BMSCs. **b**, Quantitative analysis of TRPM7 levels. The data are presented as the means ± SDs. n=3 biological replicates for each group. Unpaired two tailed test was used. **c**, Immunofluorescence of Trpm7 (green) costained with Nestin (red) in testicular sections. Scale bar, 50 μm. **d**, The functional network was constructed through GeneMANIA to predict and analyze the interaction between Trpm7 and mitochondrial transport genes. **e**, Pearson correlation analysis showed the interaction between Trpm7 and mitochondrial transport genes.Source data are provided as a Source Data file.

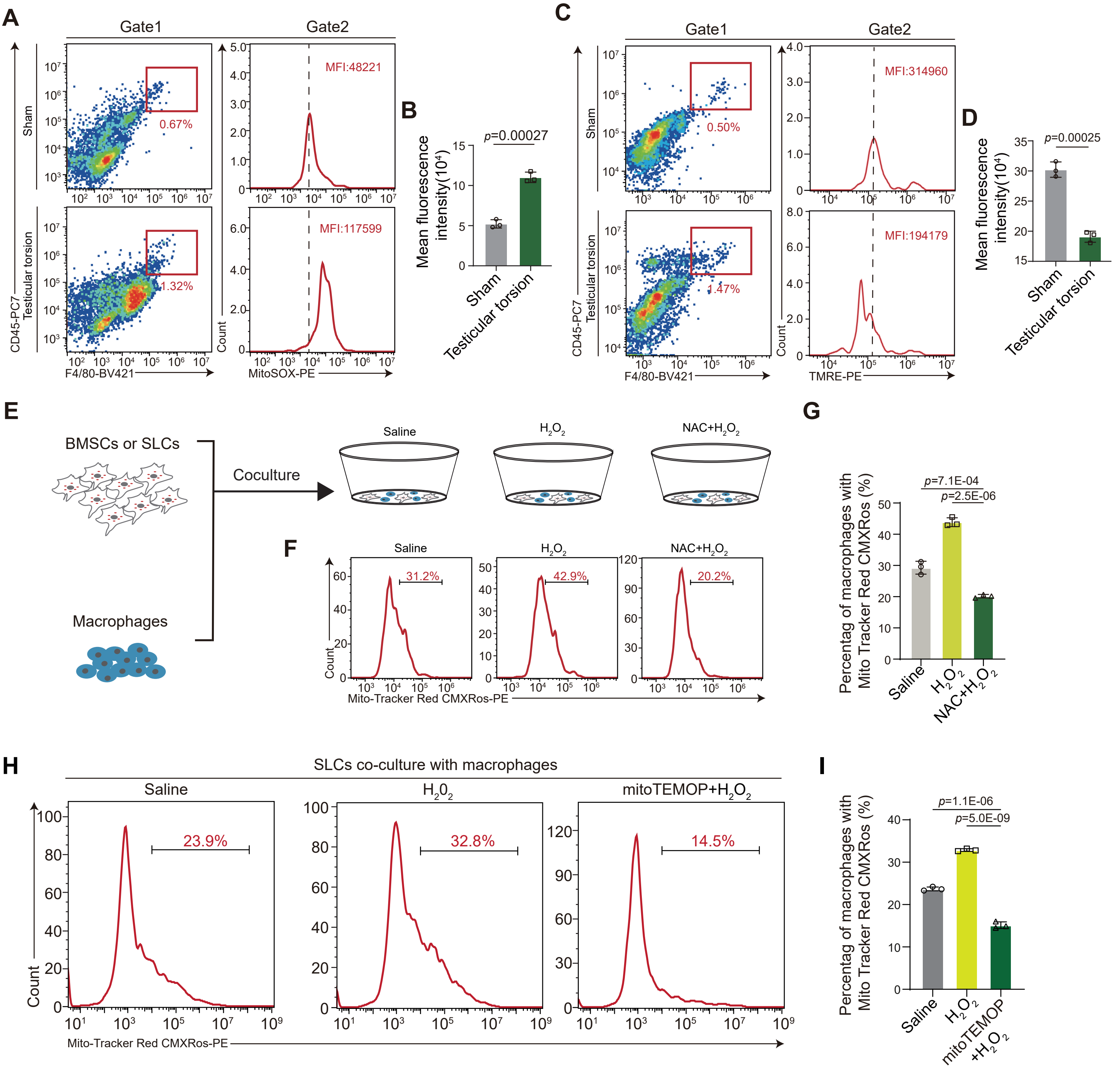
A
Gate1
Gate2
Sham
Testicular torsion
CD45-PC7
F4/80-BV421
0.67%
1.32%
MFI:48221
MFI:117599
Count
MitoSOX-PE
B
p=0.00027
Mean fluorescence intensity(10^4)
Sham
Testicular torsion
C
Gate1
Gate2
0.50%
1.47%
MFI:314960
MFI:194179
TMRE-PE
D
p=0.00025
E
BMSCs or SLCs
Coculture
Macrophages
Saline
H_2O_2
NAC+H_2O_2
F
31.2%
42.9%
20.2%
Mito-Tracker Red CMXRos-PE
G
p=7.1E-04
p=2.5E-06
Percentag of macrophages with Mito Tracker Red CMXRos (%)
H
SLCs co-culture with macrophages
23.9%
32.8%
14.5%
mitoTEMOP+H_2O_2
I
p=1.1E-06
p=5.0E-09

Supplementary Fig. 15| ROS promote mitochondrial transfer. a, Flow cytometry for detecting mitochondrial ROS levels in CD45$^+$F4/80$^+$ macrophages in sham and testicular torsion mouse testes. **b**, Quantification of mitochondrial ROS levels. The data are presented as the means ± SDs, n=3 biological replicates for each group. Unpaired two tailed Student's t test was used. **c**, Flow cytometry for detecting mitochondrial ΔΨm in CD45$^+$F4/80$^+$ macrophages in sham and testicular torsion mouse testes. **d**, Quantification of mitochondrial ΔΨm in CD45$^+$F4/80$^+$ macrophages. The data are presented as the means ± SDs. n=3 biological replicates for each group. Unpaired-tailed Student's t test was used. **e**, Schematic of the experiment in which BMSCs or SLCs were cultured with macrophages treated with H_2O_2 or NAC and H_2O_2 for 24 h. **f**, Representative flow cytometry profiles showing that SLCs transfer mitochondria to total macrophages. **g**, The percentage of mitochondrial transfer to total macrophages in each group was analyzed and graphed. The data are presented as the means ± SDs. n=3 biological replicates for each group. One-way ANOVA was used. **h**, Representative flow cytometry profiles showing that SLCs transfer mitochondria to total macrophages treated with H_2O_2 or mitoTEMOP and H_2O_2 for 24 h. **i**, The percentage of mitochondrial transfer to total macrophages in each group was analyzed and graphed. The data are presented as the means ± SDs. n=3 biological replicates for each group. One-way ANOVA was used. Source data are provided as a Source Data file.

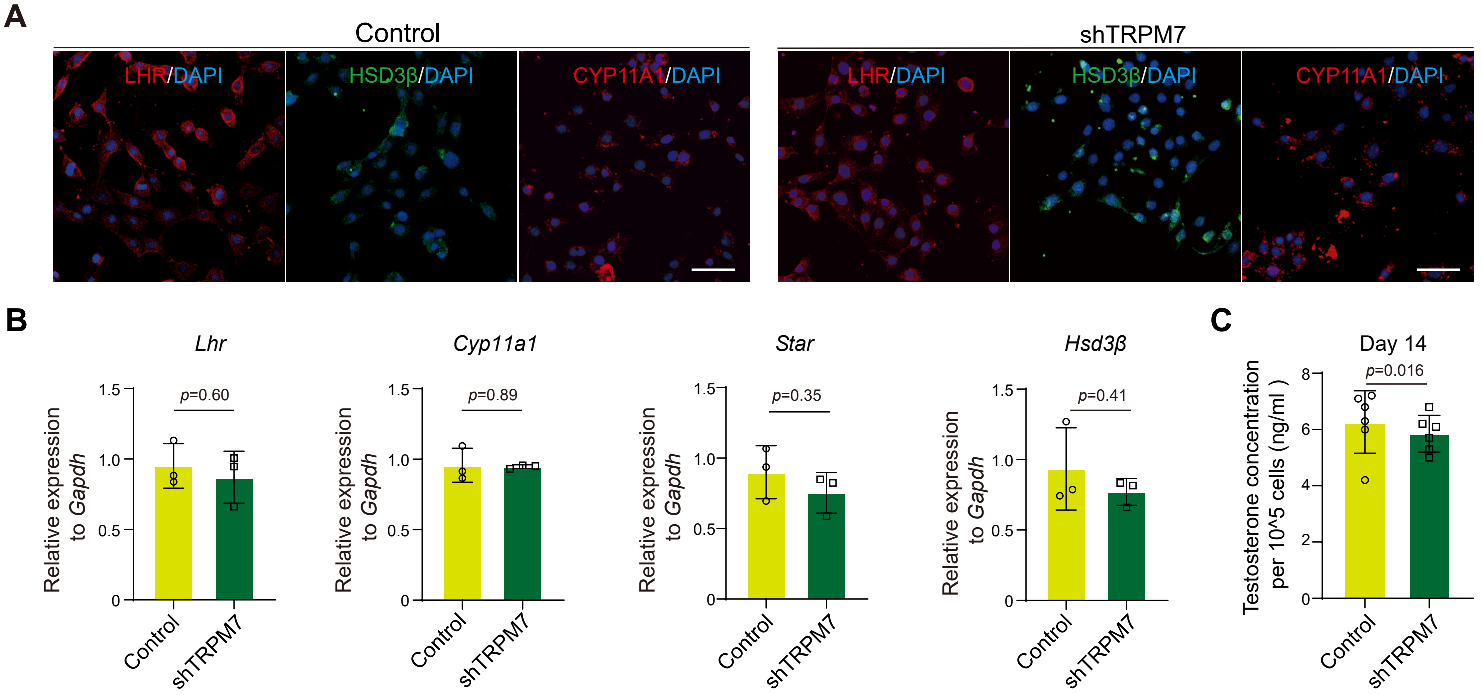
A
Control
shTRPM7
LHR/DAPI
HSD3β/DAPI
CYP11A1/DAPI
B
Lhr
Cyp11a1
Star
Hsd3β
Relative expression to Gapdh
p=0.60
p=0.89
p=0.35
p=0.41
1.5
1.0
0.5
0
Control
shTRPM7
C
Day 14
Testosterone concentration per 10^5 cells (ng/ml)
p=0.016
8
6
4
2
0

Supplementary Fig. 16| Knockdown of TRPM7 has no effect on SLCs differentiation. a, SLCs differentiation into LCs from different groups on day 14. LCs were identified as HSD3β, LHR, and CYP11A1 cells. Scale bar, 20 μm. **b**, RT–PCR analysis of the relative mRNA expression of testosterone production-related genes on day 14 during the induced differentiation of SLCs or SLCs^{shTRPM7}. **c**, Quantification of testosterone levels in the supernatants of medium during induced differentiation of primary SLCs or SLCs^{shTRPM7}. The data are presented as the means ± SDs. n = 6 biological replicates for each group. Unpaired two tailed t test was used. Source data are provided as a Source Data file.

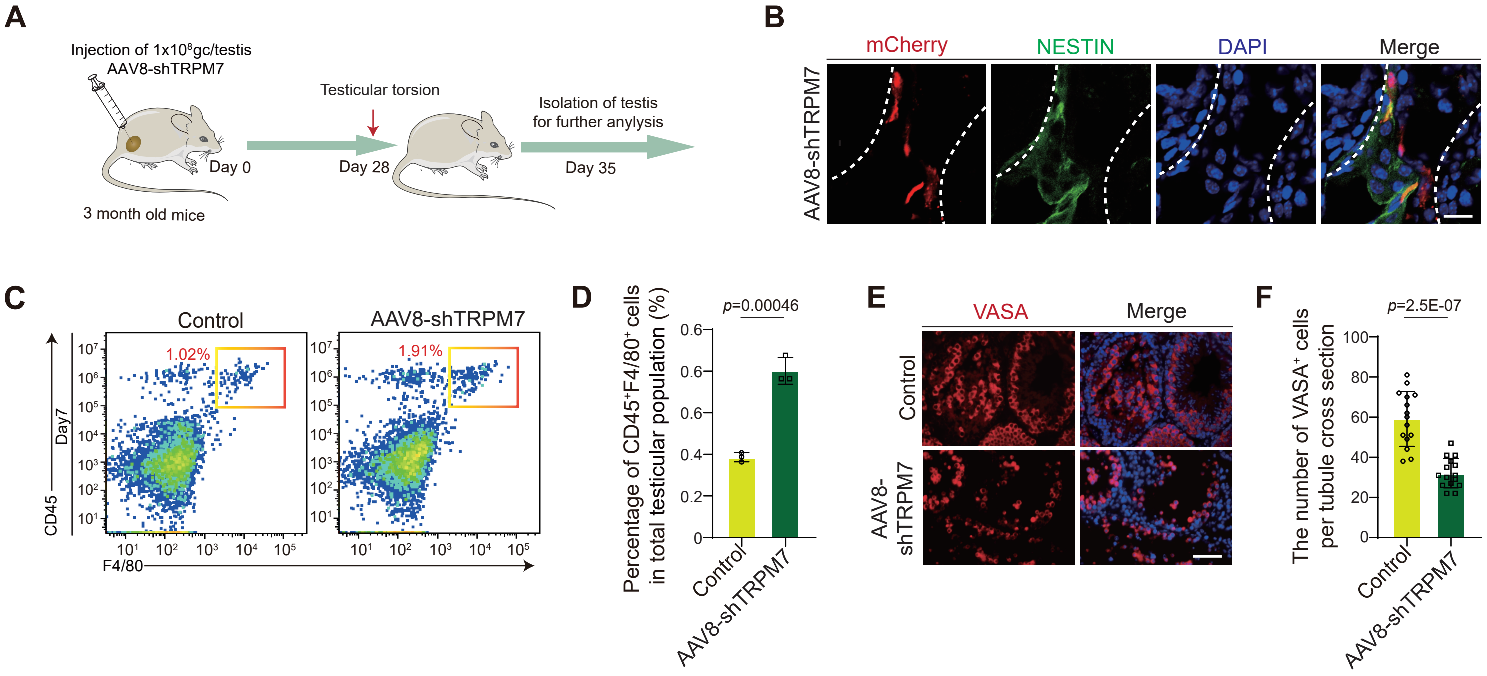

A
Injection of 1x10⁸gc/testis
AAV8-shTRPM7
Testicular torsion
Isolation of testis
for further anylysis
Day 0
Day 28
Day 35
3 month old mice
B
mCherry
NESTIN
DAPI
Merge
AAV8-shTRPM7
C
Control
AAV8-shTRPM7
1.02%
1.91%
CD45
Day7
F4/80
D
p=0.00046
Percentage of CD45⁺F4/80⁺ cells in total testicular population (%)
Control
AAV8-shTRPM7
E
VASA
Merge
Control
AAV8-shTRPM7
F
p=2.5E-07
The number of VASA⁺ cells per tubule cross section
Control
AAV8-shTRPM7

Supplementary Fig. 17| Knockdown of TRPM7 in endogenous SLCs affects tissue homeostasis after testicular torsion. **a**, Schematic illustration of AAV-shTRPM7 injection into the interstitium of the testes *in vivo*. **b**, Representative image showing the coexpression of mCherry and the SLCs marker nestin. **c**, Flow cytometry for detecting the percentage of $CD45^+F4/80^+$ macrophages among total testicular cells on day 7 after testicular torsion. **d**, Quantification of the percentage of $CD45^+F4/80^+$ macrophages. The data are presented as the means ± SDs, n=3 biological replicates for each group. Unpaired two-tailed Student's t test was used. **e**, Immunostaining of the germ cell marker VASA (red) after testicular torsion in paraffin sections. Scale bars, 50 μm. **f**, Quantitative analysis of the number of $VASA^+$ in seminiferous tubules per section. The data are presented as the means ± SDs. n=3 biological replicates for each group. Unpaired two-tailed Student's t test was used. Source data are provided as a Source Data file.

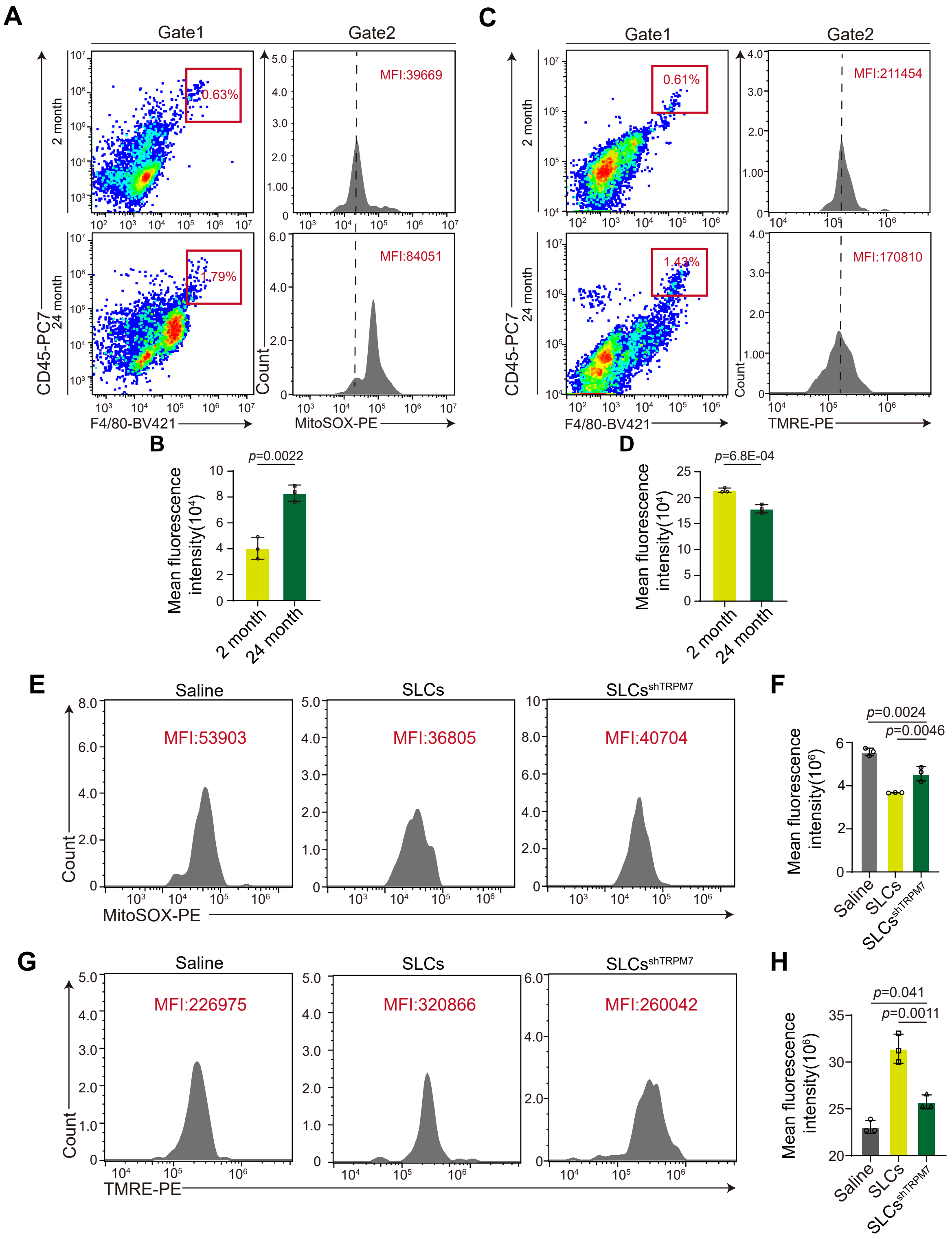
A
Gate1
Gate2
2 month
24 month
CD45-PC7
F4/80-BV421
0.63%
1.79%
MFI:39669
MFI:84051
Count
MitoSOX-PE
C
0.61%
1.43%
MFI:211454
MFI:170810
TMRE-PE
B
p=0.0022
Mean fluorescence intensity(10⁴)
D
p=6.8E-04
E
Saline
SLCs
SLCs^shTRPM7
MFI:53903
MFI:36805
MFI:40704
F
p=0.0024
p=0.0046
Mean fluorescence intensity(10⁶)
G
MFI:226975
MFI:320866
MFI:260042
H
p=0.041
p=0.0011

Supplementary Fig. 18| Mitochondrial propagation reduces macrophage ROS levels in aging mice. **a**, Flow cytometry for detecting mitochondrial ROS levels in $CD45^+F4/80^+$ macrophages from 2-month and 24-month mouse testes. **b**, Quantification of mitochondrial ROS levels. The data are presented as the means ± SDs. n=3 biological replicates for each group. Unpaired t tests were used. **c**, Flow cytometry for detecting mitochondrial $\Delta\Psi m$ in $CD45^+F4/80^+$ macrophages from 2-month and 24-month mouse testes. **d**, Quantification of mitochondrial $\Delta\Psi m$ in $CD45^+F4/80^+$ macrophages. The data are presented as the means ± SDs. n=3 biological replicates for each group. Unpaired t tests were used. **e**, Flow cytometry for detecting macrophage mitochondrial ROS levels after coculture with SLCs or $SLCs^{shTRPM7}$ in vitro. **f**, Quantification of mitochondrial ROS levels in macrophages of each group. The data are presented as the means ± SDs. n=3 biological replicates for each group. One-way ANOVA was used. **g**, Flow cytometry for detecting mitochondrial $\Delta\Psi m$ in macrophages after coculture with SLCs or $SLCs^{shTRPM7}$ in vitro. **h**, Quantification of mitochondrial $\Delta\Psi m$ in macrophages from each group. The data are presented as the means ± SDs. n=3 biological replicates for each group. One-way ANOVA was used. Source data are provided as a Source Data file.

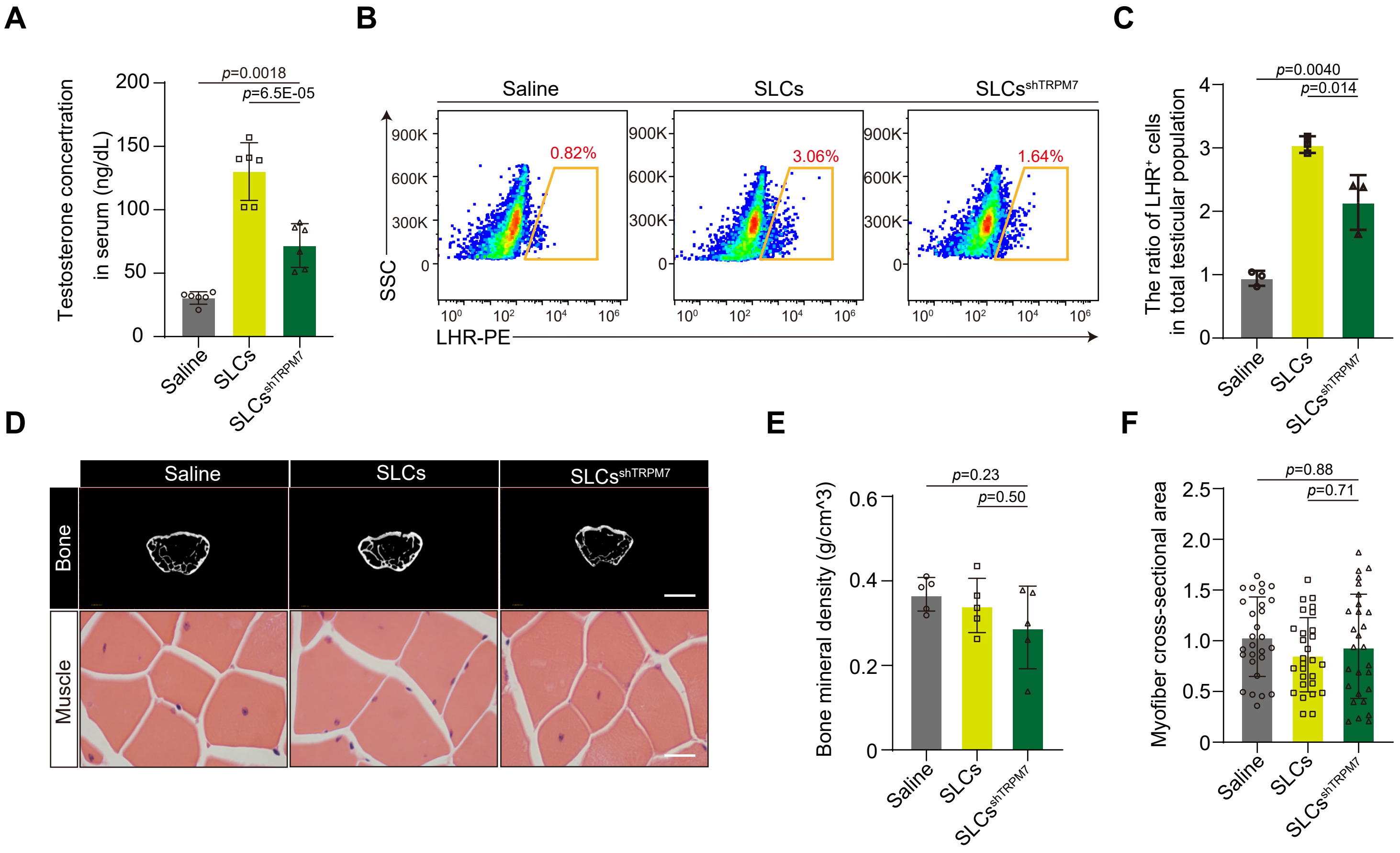
A
B
C
Testosterone concertration in serum (ng/dL)
p=0.0018
p=6.5E-05
Saline
SLCs
SLCsshTRPM7
0.82%
3.06%
1.64%
SSC
LHR-PE
The ratio of LHR+ cells in total testicular population
p=0.0040
p=0.014
D
E
F
Bone
Muscle
Bone mineral density (g/cm^3)
p=0.23
p=0.50
Myofiber cross-sectional area
p=0.88
p=0.71

Supplementary Fig. 19| Therapeutic effects of SLCs versus SLCs^{shTRPM7} on testosterone levels in testis aging. **a**, Quantitative analysis of the testosterone level in the serum of the saline-, SLCs or SLCs^{shTRPM7} group. Data are presented as the means ± SDs. n=6 biological replicates for each group. One-way ANOVA was used. **b**, Flow cytometry for detecting the percentage of LHR^{+} in total testicular cells in the saline-, SLCs or SLCs^{shTRPM7} group on day 28. **c**, Quantification analysis of the percentage of LHR^{+} cells in the total testis of the saline, SLCs and SLCs^{shTRPM7} groups. The data are presented as the means ± SDs. n=3 biological replicates for each group. **d**, Representative image showing femoral head and quadriceps muscle imaging in the saline-, SLC- and SLCs^{shTRPM7}-treated groups. **e**, Quantitative analysis of the total bone mineral density (BMD) of the femoral head in the saline, SLC and SLCs^{shTRPM7} groups. The data are presented as the means ± SDs. n=3 biological replicates for each group. One-way ANOVA was used. **f**, Quantitative analysis of the myofiber cross-sectional area of the quadriceps muscle in the saline-, SLCs and SLCs^{shTRPM7}-treated groups. The data are presented as the means ± SDs. n=3 biological replicates for each group. One-way ANOVA was used. Source data are provided as a Source Data file.

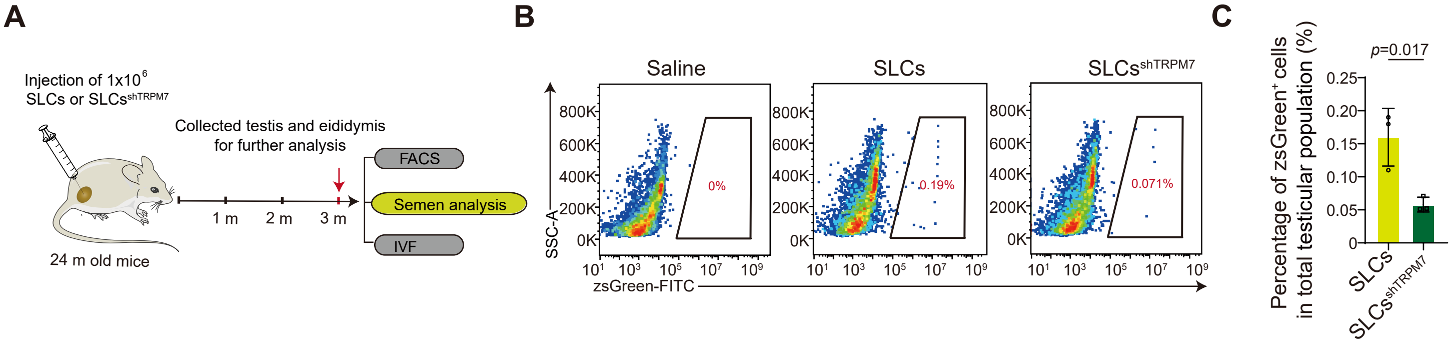

D

Mouse numbers		Saline 1	Saline 2	Saline 3	SLCs 1	SLCs 2	SLCs 3	SLCs^{shTRPM7} 1	SLCs^{shTRPM7} 2	SLCs^{shTRPM7} 3
Sperm counts (million/mL)		813	699	861	1068	1320	1272	789	546	822
Sperm motility	PR(%)	9.7%	6.8%	9.8%	14.8%	11.8%	14.9%	8%	11.6%	10.7%
	NR(%)	19.4%	18.2%	18.5%	27.6%	28.6%	31.7%	23.8%	19.4%	22.2%
	IM(%)	70.8%	75.1%	71.7%	57.6%	59.6%	53.4%	68.1%	69%	67.1%

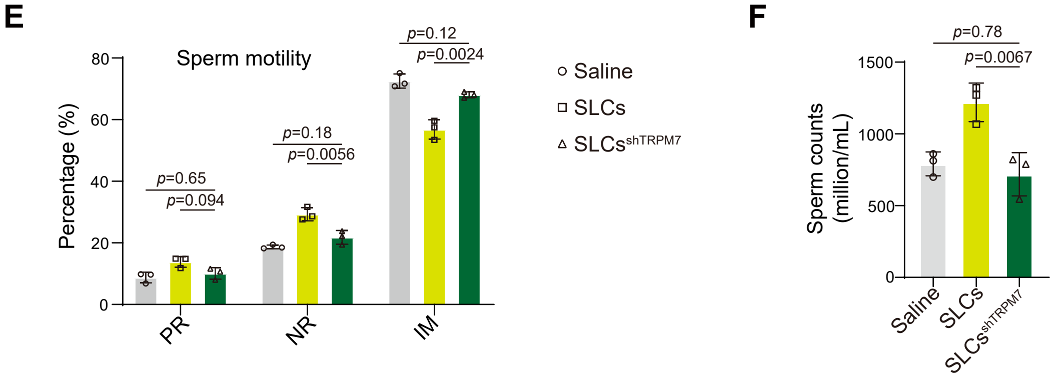

G IVF

Origin of Sperm	No. of Injected oocytes	Two-cell embryos No. (%)	Blastocysts No. (%)
Saline	110	65.45%(72)	73.61%(53)
SLCs	108	75.00%(81)	81.48%(66)
SLCs^{shTRPM7}	113	67.26 %(76)	72.37%(55)

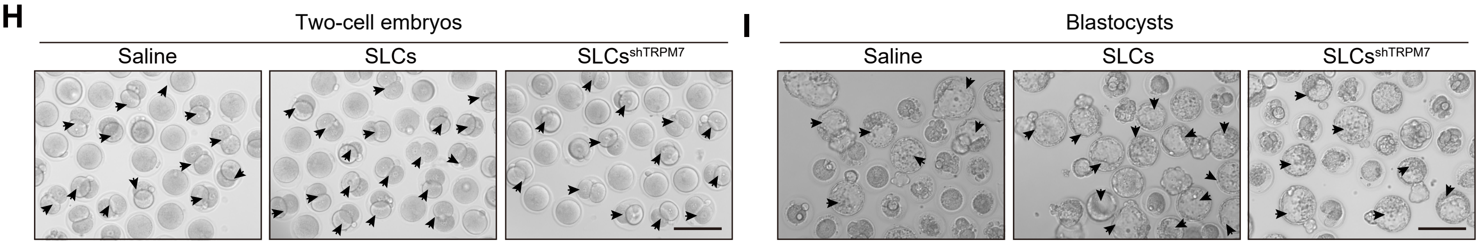

Supplementary Fig. 20| The long-term therapeutic effects of SLCs versus SLCs^{shTRPM7} on the fertility status of aging mice. **a**, Schematic illustration of SLCs^{zsGreen} or SLCs$^{shTRPM7/zsGreen}$ injection into the parenchyma of the testes in aging mice. **b**, Flow cytometry for detecting the percentage of zsGreen^{+} cells in total testicular cells after three months. **c**, Quantification analysis of the percentage of zsGreen^{+} cells in total testicular cells after three months. The data are presented as the means ± SDs. n=3 biological replicates for each group. One-way ANOVA was used. **d-f**, Sperm counts and sperm motility in the SLCs and SLCs^{shTRPM7} groups. PR: progressive motile, NR: nonprogressive motile, IM: immotilitile. The data are presented as the means ± SDs, n=3 biological replicates for each group, one-way ANOVA was used. **g**, The trilinear table shows all embryo injection, two-cell embryo and blastocyst IVF data in the sham, saline, BMSCs and SLCs groups. **h-i**, Bright field diagram of the 2-cell, blastocyst stages among the saline, SLCs and SLCs^{shTRPM7} groups. The arrows indicate normal developing embryos. Scale bar, 200 μm. Source data are provided as a Source Data file.

Gene	Forward Primer (5′-3′)	Reverse Primer (5′-3′)
m*IL-1α*	GCACCTTACACCTACCAGAGT	AAACTTCTGCCTGACGAGCTT
m*TNF-α*	CCCTCACACTCAGATCATCTTCT	GCTACGACGTGGGCTACAG
m*IL-1β*	ATGATGGCTTATTACAGTGGCAA	GTCGGAGACGTAGCTGGA
m*IFN-γ*	GCCACGGCACAGTCATTGA	TGCTGATGGCCTGATTGTCTT
m*Tslp*	ACGGATGGGGCTAACTTACAA	AGTCCTCGATTTGCTCGAACT
m*GM-CSF*	TCGTCTCTAACGAGTTCTCCTT	CGTAGACCCTGCTCGAATATCT
m*Cyp11a1*	CACTGAGACTCCACCCCATC	GGCAAAGCTAGCCACCTGTA
m*Lhr*	TTTGGCAACTTGACAGTCC	CTCCCTGTCTGCCAGTCT
m*3β-HSD*	GCCTTCGAGACCCCAAGAAG	AAGGCTCCAGCTGGCATTAG
m*Star*	CCGGAGCAGAGTGGTGTCA	CAGTGGATGAAGCACCATGC
m*Gapdh*	AGGTCGGTGTGAACGGATTTG	GGGGTCGTTGATGGCAACA

Supplementary Table S1: Primers used to amplify transcripts during RT–PCR analysis.
