# Supplementary material for: Stem Leydig cells support macrophage immunological homeostasis through mitochondrial transfer in mice
Source: Nat Commun. 2024 Mar 8;15:2120. doi: 10.1038/s41467-024-46190-2 (PMC10924100; doi:10.1038/s41467-024-46190-2)
Supplement: Supplementary file 3 — Description of Additional Supplementary Files [file 41467_2024_46190_MOESM3_ESM.pdf]

Description of Additional Supplementary Files

File Name: Supplementary Data 1

Supplementary Data1: Proteomic sequencing analysis of testicular mesenchymal exosomes showed differential expression profiles in young and aging testes related to Fig. 6a
