# Supplementary material for: Stem Leydig cells support macrophage immunological homeostasis through mitochondrial transfer in mice
Source: Nat Commun. 2024 Mar 8;15:2120. doi: 10.1038/s41467-024-46190-2 (PMC10924100; doi:10.1038/s41467-024-46190-2)
Supplement: Supplementary file 5 — Reporting Summary [file 41467_2024_46190_MOESM5_ESM.pdf]

nature portfolio

Corresponding author(s): *Double-blind peer review submissions: write DBPR and your manuscript number here instead of author names.*

Last updated by author(s): *YYYY-MM-DD*

Reporting Summary

Nature Portfolio wishes to improve the reproducibility of the work that we publish. This form provides structure for consistency and transparency in reporting. For further information on Nature Portfolio policies, see our Editorial Policies and the Editorial Policy Checklist.

Statistics

For all statistical analyses, confirm that the following items are present in the figure legend, table legend, main text, or Methods section.

n/a	Confirmed	
☐	☒	The exact sample size (*n*) for each experimental group/condition, given as a discrete number and unit of measurement
☐	☒	A statement on whether measurements were taken from distinct samples or whether the same sample was measured repeatedly
☐	☒	The statistical test(s) used AND whether they are one- or two-sided<br>*Only common tests should be described solely by name; describe more complex techniques in the Methods section.*
☒	☐	A description of all covariates tested
☒	☐	A description of any assumptions or corrections, such as tests of normality and adjustment for multiple comparisons
☐	☒	A full description of the statistical parameters including central tendency (e.g. means) or other basic estimates (e.g. regression coefficient) AND variation (e.g. standard deviation) or associated estimates of uncertainty (e.g. confidence intervals)
☐	☒	For null hypothesis testing, the test statistic (e.g. *F*, *t*, *r*) with confidence intervals, effect sizes, degrees of freedom and *P* value noted<br>*Give P values as exact values whenever suitable.*
☒	☐	For Bayesian analysis, information on the choice of priors and Markov chain Monte Carlo settings
☐	☒	For hierarchical and complex designs, identification of the appropriate level for tests and full reporting of outcomes
☒	☐	Estimates of effect sizes (e.g. Cohen's *d*, Pearson's *r*), indicating how they were calculated

Our web collection on statistics for biologists contains articles on many of the points above.

Software and code

Policy information about availability of computer code

Data collection	The RNA-seq was performed on an Illumina Novaseq platform.<br>Flow cytometric assays were performed on CytoFLEX and analysed in FlowJo
Data analysis	Main softwares: Hisat2 (v2.1.0); HTSeq (v0.11.3); bbduk (v38.18); trimmomatic (v0.39); bowtie2 (v2.3.5.1);R (v3.5.3).<br>GraphPad Prism 9.2.0, FlowJo 10.0.8, imageJ 1.8.0, CytExpert 2.4.0

For manuscripts utilizing custom algorithms or software that are central to the research but not yet described in published literature, software must be made available to editors and reviewers. We strongly encourage code deposition in a community repository (e.g. GitHub). See the Nature Portfolio guidelines for submitting code & software for further information.

Data

Policy information about availability of data

All manuscripts must include a data availability statement. This statement should provide the following information, where applicable:

- Accession codes, unique identifiers, or web links for publicly available datasets
- A description of any restrictions on data availability
- For clinical datasets or third party data, please ensure that the statement adheres to our policy

Raw sequencing data for RNA-seq of BMSCs and SLCs was retrieved from Gene Expression Omnibus (GEO) under accession number GSE254881 (https://www.ncbi.nlm.nih.gov/geo/query/acc.cgi?acc=GSE254881)

Field-specific reporting

Please select the one below that is the best fit for your research. If you are not sure, read the appropriate sections before making your selection.

☒ Life sciences ☐ Behavioural & social sciences ☐ Ecological, evolutionary & environmental sciences

For a reference copy of the document with all sections, see nature.com/documents/nr-reporting-summary-flat.pdf

Life sciences study design

All studies must disclose on these points even when the disclosure is negative.

Sample size

mice were anesthetized with sodium pentobarbital, and the right testis was rotated 720° in a clockwise direction and fixed to the scrotum with silk suture for 1 h, after which the left testis was removed. Detection was performed by untwisting the testis. The testes that returned to red from dark purple 5 min after detorsion were used in subsequent studies. Approximately 1 × 106 BMSCs or SLCs in 20 μL of saline were injected into the interstitium of the recipient testes immediately after detorsion (n = 6 mice per group), whereas control animals (n = 6 mice per group) received the same volume of saline. After surgery, the animals were kept in the original environment. Testes and serum from all animals were isolated and tested after cell treatment.

GGene delivery in animal models

AAV viral vectors were prepared by Hanbio Biotechnology (pAAV-CAG-shTRPM7-mCherry). Mice were anesthetized by i.p. injection. Using sterile surgical scissors, a single incision was made on the ventral skin and body wall approximately 1.0 cm anterior to the genitals. The testes were pulled out by holding the fat pad. Secure the testis with fine forceps, inject the AAV particles (8 × 1010 gc/testis) into the testicular interstitium using a 33-gauge needle syringe (Hamilton, Switzerland), and then suture the incision. Surgery is performed under aseptic conditions.

Data exclusions

Describe any data exclusions. If no data were excluded from the analyses, state so OR if data were excluded, describe the exclusions and the rationale behind them, indicating whether exclusion criteria were pre-established.

Replication

At least three independent biological replicates were performed.

Randomization

mice were randomly divided into groups.

Blinding

Investigators were blinded to group allocation during analysis of staining

Reporting for specific materials, systems and methods

We require information from authors about some types of materials, experimental systems and methods used in many studies. Here, indicate whether each material, system or method listed is relevant to your study. If you are not sure if a list item applies to your research, read the appropriate section before selecting a response.

Materials & experimental systems

n/a	Involved in the study	
☐	☒	Antibodies
☒	☐	Eukaryotic cell lines
☒	☐	Palaeontology and archaeology
☐	☒	Animals and other organisms
☒	☐	Human research participants
☒	☐	Clinical data
☒	☐	Dual use research of concern

Methods

n/a	Involved in the study	
☒	☐	ChIP-seq
☐	☒	Flow cytometry
☒	☐	MRI-based neuroimaging

Antibodies

Antibodies used

IF
mouse anti-DDX4 (Abcam, ab27591, 1:500, RRID:AB_11139638)
rabbit anti-SYCP3 (Abcam, ab15093, 1:400, RRID:AB_301639),
rabbit anti-α-SMA (Abcam, ab5694, 1:500, RRID:AB_91982),
rat anti-F4/80 (Abcam, ab6640, 1:200, RRID:AB_1140040),
mouse anti-3β-HSD (Santa Cruz Biotechnology, sc-515120, 1:100, RRID:AB_2721058),
rabbit anti-CYP11A1 (Cell Signaling, 14217, 1:400, RRID:AB_2631970),
rabbit anti-Myosin IIa (Cell Signaling, 49349, 1:400),
rabbit anti-TRPM7 (Affinity, DF7513, 1:400, RRID: AB_2841012),
mouse anti-Nestin (Abcam, ab134107, 1:400, RRID:AB_2841012).

goat anti-mouse Alexa Fluor 488 (Invitrogen, A28175, 1:40, RRID:AB_2536161),
goat anti-rabbit Alexa Fluor 488 (Abcam, ab150077, 1:400, RRID:AB_2630356),
goat anti-rabbit Alexa Fluor 555 (Abcam, ab150078, 1:400, RRID:AB_2722519),
goat anti-rat Alexa Fluor 488 (Abcam, ab150165, 1:400, RRID:AB_2650997).
FACS
CD51-BV421 antibody (BD Bioscience, 740062, 1:100 RRID:AB_2739827),
F4/80-BV421 antibody (BioLegend, 123137, 1:10, RRID:AB_2563102),
CD45-PE/CY7 antibody (BioLegend, 103113, 1:10, RRID:AB_312979),
CD11b-FITC antibody (BioLegend, 101205, 1:100, RRID:AB_312789),
Ly6C-APC antibody (BioLegend, 128015, 1:100, RRID:AB_1732087),
Ly6G-APC antibody (BioLegend, 127613, 1:100, RRID:AB_1877163),
CD86-FITC antibody (BioLegend, 105109, 1:100, RRID:AB_313163),
CD206-FITC antibody (BioLegend, 141703, 1:100, RRID:AB_10901166),
Agr1-PE antibody (R&D Systems, IC5868P, 1:100),
INOS-PE antibody (BioLegend, 128015, 1:100, RRID:AB_2876745),
anti-LHR (Alomone labs, ALR-010, 1:100,RRID:AB_2340988),
donkey anti-rabbit IgG-PE (BioLegend, 406421, 1:100).
wb
anti-tubulin (Abcam, ab59680,1:10,000, RRID:AB_2210403)
rabbit anti-TRPM7 (Affinity, DF7513, 1:400, RRID: AB_2841012)
anti-mouse HRP (ZSJB-BIO, zb2305, 1:1000, RRID:AB_2747415)
anti-rabbit HRP (ZSJB-BIO, zb2301, 1:1000, RRID:AB_2747412).

Validation

Validation information was provided by manufacturer.
https://www.abcam.com/products/primary-antibodies/ddx4--mvh-antibody-mabcam27591-ab27591.html
https://www.abcam.com/products/primary-antibodies/scp3-antibody-ab15093.html
https://www.abcam.com/products/primary-antibodies/alpha-smooth-muscle-actin-antibody-ab5694.html
https://www.abcam.com/products/primary-antibodies/f480-antibody-cia3-1-macrophage-marker-ab6640.html
https://www.scbt.com/zh/p/3beta-hsd-antibody-a-1
https://www.cellsignal.cn/products/primary-antibodies/cyp11a1-d8f4f-rabbit-mab/14217
https://www.cellsignal.cn/products/primary-antibodies/myosin-iia-e7y9o-rabbit-mab/49349
https://affbiotech.cn/goods-11277-DF7513-TRPM7_Antibody.html
https://www.abcam.cn/products/primary-antibodies/nestin-antibody-ab134017.html
https://www.thermofisher.cn/cn/zh/antibody/product/Goat-anti-Mouse-IgG-H-L-Secondary-Antibody-Recombinant-Polyclonal/A28175
https://www.abcam.cn/products/secondary-antibodies/goat-rabbit-igg-hl-alexa-fluor-488-ab150077.html
https://www.abcam.cn/products/secondary-antibodies/goat-rabbit-igg-hl-alexa-fluor-555-ab150078.html
https://www.abcam.cn/products/secondary-antibodies/goat-rat-igg-hl-alexa-fluor-488-preadsorbed-ab150165.html
https://www.bdbiosciences.com/zh-cn/products/reagents/flow-cytometry-reagents/research-reagents/single-color-antibodies-ruo/bv421-rat-anti-mouse-cd51.740062
https://www.biolegend.com/en-us/explore-new-products/brilliant-violet-421-anti-mouse-f4-80-antibody-7199?GroupID=BLG5319
https://www.biolegend.com/en-gb/products/pe-cyanine7-anti-mouse-cd45-antibody-1903?GroupID=BLG1932
https://www.biolegend.com/en-gb/products/fitc-anti-mouse-human-cd11b-antibody-347?GroupID=BLG10660
https://www.biolegend.com/en-us/global-elements/pdf-popup/apc-anti-mouse-ly-6c-antibody-6047?GroupID=BLG7242
https://www.biolegend.com/en-gb/products/purified-anti-mouse-ly-6g-antibody-4767?GroupID=BLG7232
https://www.biolegend.com/en-us/search-results/fitc-anti-mouse-cd86-antibody-1965?GroupID=BLG10719
https://www.biolegend.com/fr-fr/products/fitc-anti-mouse-cd206-mmr-antibody-7318?GroupID=BLG9506
https://www.rndsystems.com/cn/products/human-mouse-rat-arginase-1-arg1-antibody_af5868
https://www.biolegend.com/en-gb/products/pe-anti-nos2-inos-antibody-19910
https://www.alomone.com/p/anti-lh-receptor-extracellular/ALR-010
https://www.biolegend.com/en-gb/products/pe-donkey-anti-rabbit-igg-minimal-x-reactivity-9751?GroupID=BLG3472
https://www.abcam.cn/products/primary-antibodies/tubulin-antibody-loading-control-ab59680.html
https://affbiotech.cn/goods-11277-DF7513-TRPM7_Antibody.html
http://www.zsbio.com/product/ZB-2305
http://www.zsbio.com/product/ZB-2301

Animals and other organisms

Policy information about studies involving animals; ARRIVE guidelines recommended for reporting animal research

Laboratory animals

C57BL/6J mice (male, 8-10 weeks old) were purchased from the Animal Center at the Medical Laboratory of Guangdong Province. Actin-zsGreen mice were purchased from GuangDong GemPharmatech Co., Ltd.

Wild animals

This study didn't involve wild animals.

Field-collected samples

This study didn't involve field-collected samples.

Ethics oversight

All experimental procedures involving animals were in accordance with the guidelines of the Animal Care and Use Committee of the First Affiliated Hospital of Sun Yat-sen University (2021000022) .All animal experiments abide by the ARRIVE guidelines.

Note that full information on the approval of the study protocol must also be provided in the manuscript.

Flow Cytometry

Plots

Confirm that:

☒ The axis labels state the marker and fluorochrome used (e.g. CD4-FITC).

☒ The axis scales are clearly visible. Include numbers along axes only for bottom left plot of group (a 'group' is an analysis of identical markers).

☒ All plots are contour plots with outliers or pseudocolor plots.

☒ A numerical value for number of cells or percentage (with statistics) is provided.

Methodology

Sample preparation	Testicular cells derived from C57BL/6 mice at postnatal day 7 or 8-10 weeks were minced, incubated with 1 mg/mL collagenase type IV at 37°C for 15 min, centrifuged at 1500 × g for 5 min at room temperature, and filtered using staining buffer (PBS with 0.5% BSA) through a 70 µm cell strainer to obtain a single cell suspension. Depending on the experimental design, fixation and permeabilization were performed as stated above. Blood samples were resuspended in 10 mL of red blood cell lysis buffer and incubated at 4°C for 10 min. Then, the samples were centrifuged at 1200 × g for 5 min at room temperature and filtered using staining buffer (PBS with 0.5% BSA) through a 70 µm cell strainer to obtain a single cell suspension.
Instrument	All flow cytometric assays were performed on CytoFLEX (Beckman)
Software	CytExpert 1.2.11.0 (Beckman) was used to analysis FACS date
Cell population abundance	The FACS experiments in this paper do not involve cell purity
Gating strategy	Cell debris was first discarded on the basis of FSC-A and SSC. For apoptosis, we consider APC+/ PI+ cells as late apoptotic cells and APC+/PI- as early apoptotic cells. we consider LHR+ cells are considered as Leydig cells, F480+ CD45+ cells are considered as macrophages, CD45+F480+ CD206+ or CD45+F480+ Arg1+ cells are considered as anti-inflammatory macrophages, CD45 +F480+ CD86+ or CD45+F480+ iNOS+ cells are considered as pro-inflammatory macrophages, CD11b+ Ly6C+ are considered as monocyte, CD11b+ Ly6G+ are considered neutrophil, SLCszsGreen cell are positive for FITC, cells transfected wtih mito-desRed virus are positive for PE, macrophage F480+ CD45+) that acquire exogenous mitochondria are positive for PE.

☒ Tick this box to confirm that a figure exemplifying the gating strategy is provided in the Supplementary Information.
